# A single cell atlas of human cornea that defines its development, limbal progenitor cells and their interactions with the immune cells

**DOI:** 10.1016/j.jtos.2021.03.010

**Published:** 2021-07

**Authors:** Joseph Collin, Rachel Queen, Darin Zerti, Sanja Bojic, Birthe Dorgau, Nicky Moyse, Marina Moya Molina, Chunbo Yang, Sunanda Dey, Gary Reynolds, Rafiqul Hussain, Jonathan M. Coxhead, Steven Lisgo, Deborah Henderson, Agatha Joseph, Paul Rooney, Saurabh Ghosh, Lucy Clarke, Che Connon, Muzlifah Haniffa, Francisco Figueiredo, Lyle Armstrong, Majlinda Lako

**Affiliations:** a Biosciences Institute, Faculty of Medical Sciences, Newcastle University, UK; b Newcastle Cellular Therapies Facility, Newcastle University and Newcastle Upon Tyne Hospitals NHS Foundation Trust, UK; c Department of Genetics and Developmental Biology, The Ruth and Bruce Rappaport Faculty of Medicine, Technion - Israel Institute of Technology, Israel; d NHS Blood and Transplant Tissue and Eye Services, Liverpool, UK; e Sunderland Eye Infirmary, South Tyneside and Sunderland NHS Foundation Trust, Sunderland, UK; f UK Department of Ophthalmology, Royal Victoria Infirmary and Newcastle University, Newcastle, UK

**Keywords:** Embryonic and fetal eye, Cornea, Conjunctiva, Ocular surface, Single cell RNA-Seq, Single cell ATAC-Seq, Limbal stem cells (LSCs), Limbal progenitor cells (LPCs), Limbal epithelial cells (LECs), LSCs dysplasia, Keratoconus, Limbal epithelial expansion

## Abstract

**Purpose:**

Single cell (sc) analyses of key embryonic, fetal and adult stages were performed to generate a comprehensive single cell atlas of all the corneal and adjacent conjunctival cell types from development to adulthood.

**Methods:**

Four human adult and seventeen embryonic and fetal corneas from 10 to 21 post conception week (PCW) specimens were dissociated to single cells and subjected to scRNA- and/or ATAC-Seq using the 10x Genomics platform. These were embedded using Uniform Manifold Approximation and Projection (UMAP) and clustered using Seurat graph-based clustering. Cluster identification was performed based on marker gene expression, bioinformatic data mining and immunofluorescence (IF) analysis. RNA interference, IF, colony forming efficiency and clonal assays were performed on cultured limbal epithelial cells (LECs).

**Results:**

scRNA-Seq analysis of 21,343 cells from four adult human corneas and adjacent conjunctivas revealed the presence of 21 cell clusters, representing the progenitor and differentiated cells in all layers of cornea and conjunctiva as well as immune cells, melanocytes, fibroblasts, and blood/lymphatic vessels. A small cell cluster with high expression of limbal progenitor cell (LPC) markers was identified and shown via pseudotime analysis to give rise to five other cell types representing all the subtypes of differentiated limbal and corneal epithelial cells. A novel putative LPCs surface marker, GPHA2, expressed on the surface of 0.41% ± 0.21 of the cultured LECs, was identified, based on predominant expression in the limbal crypts of adult and developing cornea and RNAi validation in cultured LECs. Combining scRNA- and ATAC-Seq analyses, we identified multiple upstream regulators for LPCs and demonstrated a close interaction between the immune cells and limbal progenitor cells. RNA-Seq analysis indicated the loss of *GPHA2* expression and acquisition of proliferative limbal basal epithelial cell markers during *ex vivo* LEC expansion, independently of the culture method used. Extending the single cell analyses to keratoconus, we were able to reveal activation of collagenase in the corneal stroma and a reduced pool of limbal suprabasal cells as two key changes underlying the disease phenotype. Single cell RNA-Seq of 89,897 cells obtained from embryonic and fetal cornea indicated that during development, the conjunctival epithelium is the first to be specified from the ocular surface epithelium, followed by the corneal epithelium and the establishment of LPCs, which predate the formation of limbal niche by a few weeks.

**Conclusions:**

Our scRNA-and ATAC-Seq data of developing and adult cornea in steady state and disease conditions provide a unique resource for defining genes/pathways that can lead to improvement in *ex vivo* LPCs expansion, stem cell differentiation methods and better understanding and treatment of ocular surface disorders.

## Graphical abstract

Schematic presentation of main techniques and findings presented in this manuscript.

**Figure:**
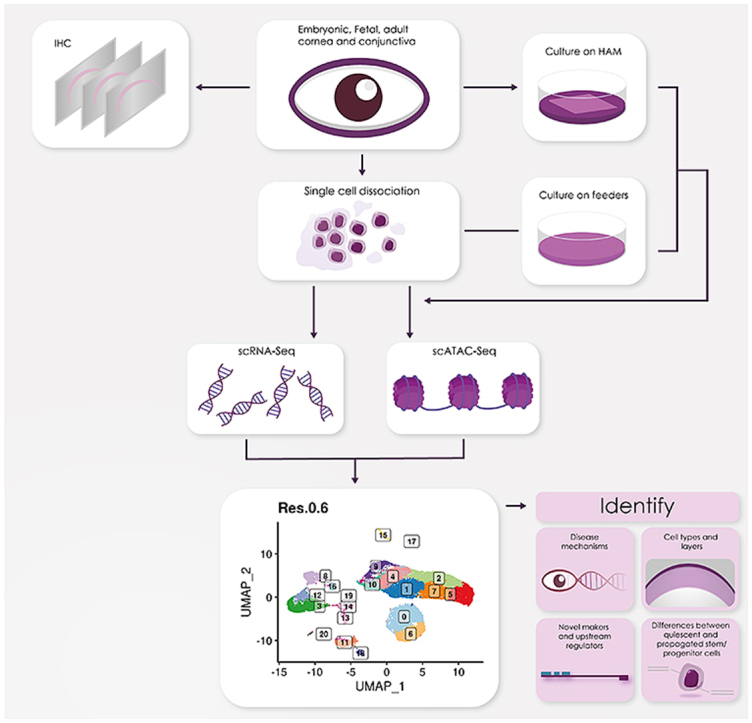

## Key findings

• scRNA-Seq of adult human cornea and conjunctiva reveals the signature of various ocular surface cell populations
• scRNA-Seq of human developing cornea identifies stage-specific definitions of corneal epithelial, stromal, and endothelial layers
• scRNA-Seq analysis results in identification of novel putative markers for LPCs (GPHA2)
• Combined scRNA- and ATAC-Seq analyses reveal key transcriptional networks in LPCs and close interactions with immune cells
• Expansion of limbal epithelium results in downregulation of GPHA2 and acquisition of proliferative limbal basal epithelial cell markers
• scRNA-Seq of keratoconus corneas reveals activation of collagenase in the corneal stroma and a reduced pool of limbal suprabasal cells
• All scRNA-Seq data are available for interactive queries via the cell browser: http://retinalstemcellresearch.co.uk/CorneaCellAtlas

## Introduction

Cornea is the transparent front part of the eye, which together with the lens focuses the light onto retina for visual processing [1]. Corneal blindness is the 2nd main cause of blindness worldwide accounting for 23 million people, adding a huge burden to health care resources [[2], [3], [4]]. Often the only treatment is surgical transplantation of donor cornea, a therapeutic option that has been in practice for more than a century. In Europe, over 40,000 blind people are waiting for corneal transplant every year [5]. This worldwide shortage of corneas results in about 10 million untreated patients globally and 1.5 million new cases of blindness annually [4].

Despite efforts to develop corneal substitutes, surgery with allogeneic donor tissue from cadavers has remained the gold standard for more than a century [1]. Transplantation of limbal stem cells (LSCs) and closely related epithelial cells have been used for corneal cell replacement therapies [6,7]. However, these clinical techniques only address epithelial regeneration, and not restoration of a dysfunctional corneal stroma or endothelium. Clearly, there is an unmet need for the design of new smart biomaterials and stem cell therapies to create a whole cornea that is indistinguishable from the original native tissue and fulfills the natural functions of a transparent cornea. This can be achieved by understanding the physical and cellular structure of the tissue under normal steady state and disease conditions.

The cornea is comprised of five layers: the outermost epithelium, Bowman's layer, the stroma, the Descemet's membrane and the endothelium [8]. The stratified epithelium covers the outermost surface of the cornea and is divided from stroma by Bowman's layer, a smooth, acellular layer made up of collagen fibrils and proteoglycans, which helps the cornea to maintain its shape. There is high corneal epithelial cell turnover due to blinking as well as physical and chemical environmental insults. The renewal of corneal epithelium is sustained by the limbal stem cells (LSCs), which are located at the Palisades of Vogt at the limbal region that marks the transition zone between clear cornea and conjunctiva [9].

The corneal stroma occupies 90% of the corneal thickness [10]. The stroma is composed of water, proteoglycans, and collagen fibrils, arranged in lamellae to reduce light scattering and enable corneal transparency. The stroma is populated by scarcely distributed keratocytes, which secrete the collagens and proteoglycans, in addition to a small population of corneal stromal stem cells (CSSCs), which are localized in the anterior peripheral (limbal) stroma near to LSCs. CSSCs display properties of mesenchymal stem cells, including clonal growth, multipotent differentiation, expression of stem cell-specific markers, ability to divide extensively *in vitro* and to generate adult keratocytes [11]. Genesis of corneal endothelium begins when periocular neural crest cells migrate between the presumptive corneal epithelium and lens vesicle and undergo a mesenchymal-to-endothelial transition to form a monolayer that occupies the posterior surface of the cornea [12]. A major function of corneal endothelium is to maintain corneal transparency by regulating corneal hydration. The corneal endothelium comprises a single layer of closely interdigitating hexagonal cells, which secrete the Descemet membrane, a cell-free matrix that mostly consists of collagens. Unlike the corneal epithelial cells, endothelial cells in humans are not endogenously renewed or replaced during a lifetime and their cell density declines at an average of approximately 0.6% per year in normal corneas throughout human life [13].

To better understand the complexity of human ocular surface, we performed single cell (sc) analyses of human cornea and adjacent conjunctiva during human development and in adulthood, in steady state and disease conditions. A similar approach was applied to the ocular anterior segment in mouse, leading to identification of a novel marker for stem and early transit amplifying cells, TXNIP [14]. While this work was under review, two studies reported the single cell transcriptomic analysis of human limbal basal epithelium [15] and murine corneal non-myelinating Schwann cells [16]. Our study complements and expands these published data by providing a comprehensive single cell atlas of *the whole* developing and adult human cornea and adjacent conjunctiva that defines their development, limbal progenitor cells and their interactions with immune cells.

## Results

### scRNA-seq of adult human cornea and adjacent conjunctiva reveals the presence of progenitor and differentiated cells in the epithelial, stromal, and endothelial layers

Human adult cornea and the adjacent conjunctiva were excised from four deceased donor eyes (51, 74, 77 and 83 years old) and dissociated to single cells. Approximately 10,000 cells were captured from each sample using the 10x Chromium Single Cell 3’ Library & Gel Bead Kit (version 3). 21,343 cells were obtained from the four adult corneas after data integration and doublet cell exclusion. These were embedded using Uniform Manifold Approximation and Projection (UMAP) and clustered using Seurat graph-based clustering, which revealed the presence of 21 cell clusters (Fig. 1A). Cluster identification was performed based on marker genes (Figure S1, Table S1), bioinformatic data mining and immunohistochemical (IF) analysis.

**Fig. 1:**
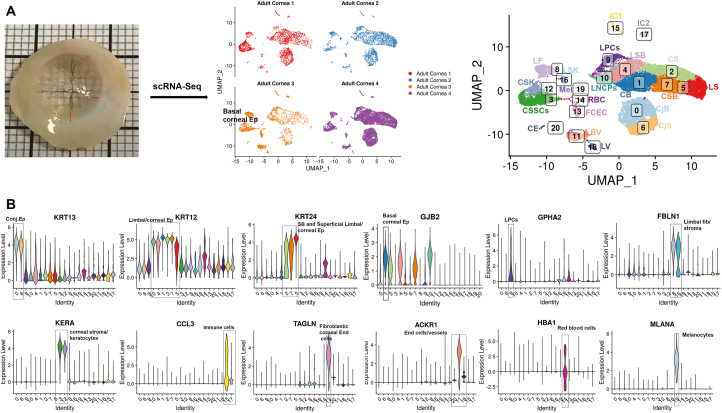
scRNA-Seq analysis of adult human cornea and conjuctiva (see also Figures S1-S12 and Table S1). A) Microphotograph of adult human ocular surface before scRNA-Seq analysis (left panel) and UMAPs of each adult human cornea and conjunctiva (middle panel). Following data integration and analysis, an integrated UMAP revealing the presence of 21 cell clusters was generated (right panel); B) Violin plots showing the presence of key markers for stem, progenitor and differentiated cells in the epithelial, stromal, and endothelial compartments. *Abbreviations for panel 1A*: BV – blood vessels, CB – corneal basal epithelium, CS – corneal superficial epithelium, CSB – corneal suprabasal epithelial cells, CjB – conjunctival basal epithelium, CjS – conjunctival superficial epithelium, CE-corneal endothelium, CSK – corneal stroma keratocytes, CSSCs – corneal stromal stem cells, FCEC – fibroblastic corneal endothelial cells, IC1 - immune cells 1, IC2 – immune cells 2, LF – limbal fibroblasts, LSB – limbal suprabasal epithelial cells, LSK – limbal stroma keratocytes, LNCPs – limbal neural crest progenitors, LPCs –limbal progenitor cells, LS – limbal superficial epithelium, LV – lymphatic vessels, Mel - melanocytes, RBC – red blood cells, *Additional abbreviations for panel 1B*: Conj– conjunctival Ep-epithelium, Fib-fibroblasts, End-endothelial.

#### Identification of epithelial cell populations

Clusters 0 and 6 displayed high expression of Keratin 13 (*KRT13*) and 19 (*KRT19*) [17,18] as well as S100A8 and S100A9 [19], which pointed to conjunctival cell fates (Fig. 1B and Figure S2A). Differential gene expression analysis indicated higher expression of *KRT6A, KRT14* and *KRT15* in cell cluster 0 compared to cell cluster 6 (Figure S2B), suggestive of a basal conjunctival epithelium, which was confirmed by IF (Figure S2C). Cluster 6 displayed higher expression of Mucin 4 (*MUC4*) and 1 (*MUC1*) as well as *KRT4*, indicative of a superficial conjunctival epithelium, which was also corroborated by IF (Figure S2C).

Cell clusters 1, 2, 4, 5, 7 and 10 displayed high expression of keratin 12 (*KRT12*), whose expression is associated with corneal and limbal epithelium [20] (Fig. 1B). Cell cluster 1 displayed high expression of gap junction protein beta 6 (*GJB2*), which is associated with a basal corneal epithelial phenotype [21]. *HES1* and *HES5*, whose expression is confined to the basal and immediate suprabasal corneal epithelium [22], were also highly expressed in cluster 1 (Table S1) and thus we defined cluster 1 as corneal basal epithelial cells. Clusters 2, 5 and 7 displayed high expression of *KRT24*, whose expression is found in the suprabasal (also known as wing cells) and the superficial layers of corneal epithelium. Amphiregulin (*AREG)* was the most highly expressed gene in cluster 2 (Figure S1, S3A). Its expression was confined to the most superficial part of corneal epithelium, (Figure S3B), leading to definition of this cluster as corneal superficial epithelium. Differential gene expression analysis indicated *LYPD2* to be highly expressed in cluster 5 (Figure S3A); hence, IF with antibody raised against LYPD2 was carried out revealing low expression in the corneal epithelium (Figure S3C), but high expression in the limbal superficial epithelium, defining cluster 5 as limbal superficial epithelium (Figure S3C’). High expression of *KRT3* and *KRT12* with *KRT24* in cell cluster 7 indicates a central cornea suprabasal or superficial epithelial cell fate. IF analysis revealed KRT3 immunostaining in the suprabasal cells and its absence from the very flat squamous cells at the corneal superficial epithelium (Fig. S3D); hence, this cluster was defined as corneal suprabasal epithelial cells.

Cluster 4, 9 and 10 were transcriptionally similar to each other (Figure S1); thus we performed differential gene expression and violin plot analyses, which revealed expression of *KRT14* and *TP63* in all three clusters; however *KRT15,* a limbal progenitor marker [23,24] was highly expressed in clusters 9 and 4 but not in cluster 10 (Figs. S4A–D). Through the differential gene expression analysis (Figure S4A), we were able to identify a unique marker for cluster 10, *CPVL*, which was not expressed in the other epithelial clusters (Fig. S5A). CPVL immunopositive cells were found in the limbal stroma next to the KRT15+ limbal basal epithelial cells, with a small minority co-expressing ΔNp63 (Figs. S5B–D). This cluster also showed high expression of *PAX6* (Table S1), a transcription factor expressed in the limbal niche cells in the stroma [25], whose function is to maintain the phenotype of neural crest progenitors. In view of this, we speculated that cluster 10 may represent the limbal neural crest derived progenitors (LNCPs). IF analysis revealed a large overlap in expression between the neural crest marker, MITF and the cluster 10 specific marker, CPVL (Figure S5E, E’), confirming the LNCPs nature of this cell population.

Cluster 4 displayed similar *KRT15* expression to cluster 9; however, this cluster was characterised by lower *KRT14* and *TP63* expression (Table S1). This cluster also expressed the differentiation marker connexin 43 (*GJA1,* Figure S4E) [26] as well as the tight junction transmembrane Claudin 1 (*CLDN1*) and 4 (*CLDN4*), which are present throughout the cell layers of corneal and conjunctival epithelium [27], suggesting that the cells in this cluster are distinct from the limbal progenitor cells (LPCs). A dual plot gene expression heatmap showed the highest co-expression of *KRT15* and *CLDN4* in cell cluster 4 and to a lesser extent in cluster 0 (Figure S6A). IF with both antibodies showed the highest co-expression of these two markers in the suprabasal cells of the limbal crypt (Figure S6B), defining this cluster as limbal suprabasal cells.

Cluster 9 showed an interesting transcriptional profile, exhibiting high expression of putative LPC markers including *CXCL14, CEBPD, TP63,* S100A2 and the more recently discovered marker, *TXNIP* [14] (Table S1), defining this cluster as LPCs. We selected cluster 9 to be at the start of the tree position in our pseudotime analysis and showed that these cells gave rise to the differentiated corneal and limbal epithelial cell clusters in accordance with its LPCs definition above (Figure S7).

#### Identification of fibroblasts and stromal cell populations

Fibulin 1 (*FBLN1*), which forms part of the extracellular matrix (ECM) that regulates the LSCs niche [28], was highly expressed in clusters 8 and 16 (Fig. 1). IF analysis, showed clear and high *FBLN1* expression under the limbal crypts (Figure S8A-C); however, this was not localised to the keratocytes marked by *CD34* expression (Figure S8B), hence, we defined cluster 8 as limbal fibroblasts. Cluster 16 displayed high expression of collagen 1 A1 (*COL1A1*) and A2 (*COL1A2*) genes, which are known to be expressed in the corneal stroma [29], but more importantly this cluster also showed high expression of collagen 3A1 (*COL3A1*) (Table S1), whose expression is found in limbal stroma [28]. A differential gene expression analysis between clusters 8 and 16, identified Osteoglycin (*OGN*) to be predominantly expressed in cluster 16 (Figure S8D, E). IF analysis (Figure S8F-H) showed a clear and distinct expression of OGN in the limbal, but not central cornea, hence we defined cluster 16 as limbal stromal keratocytes.

High expression of Keratocan (*KERA*), encoding the keratan sulfate proteoglycan that is involved in corneal transparency [30], was found in cluster 3 and 12 (Fig. 1B, S1). Differential gene expression analysis revealed Matrix Metallopeptidase 3 (*MMP3*) expression in cluster 3, but not 12 (Figure S9A). IF localised the MMP3 immunopositive cells to the stroma of peripheral and central cornea (Figure S9B-D). All the MMP3-immunostained cells also showed expression of CD105 (Figure S9E, E’), a marker of CSSCs [31], leading us to define cluster 3 as CSSCs. Since Keratocan and Lumican (*LUM*), were amongst the top ten expressed genes in cluster 12, we performed IF for Lumican, showing a clear “stacked arrangement” typical of keratocytes, which secrete the stroma extracellular matrix (Figure S9F). In view of these data we assigned cluster 12 as central stroma keratocytes.

#### Identification of immune cells

Clusters 15 and 17 were distinguished by the high expression of chemokine ligands (*CCL3* in cluster 15 and *CCL5* in cluster 17) and were defined as immune cells I and II respectively (Fig. 1, S1). Nonetheless both populations seem to have a mixed immune cell phenotype; hence, to get better insights into cell fate of these two clusters, further subclustering was performed (Figure S10A), revealing the presence of monocyte derived macrophages and dendritic cells, two subtypes of CD8 T cells and two subtypes of macrophages (Table S2, Figure S10B-G), consistent with innate cell immune profiling in cornea and conjunctiva [32,33].

#### Identification of endothelial cell populations

High expression of Transgelin (*TAGLN*, also known as *SM22α*; Fig. 1B) and Actin Alpha 2 (*ACTA2*; Figure S1) were found in cluster 13. Both markers are associated with endothelial to mesenchymal transition, which occurs during *ex vivo* expansion of corneal endothelial cells [34]*,* resulting in acquisition of a fibroblast cell phenotype [34]. Damage to the corneal endothelium in rabbits results in infiltration of leukocytes and loss of endothelial cells, which triggers morphological changes of surrounding endothelial cells and decreased collagen IV synthesis coupled to increased synthesis of collagens I and III [35]. High levels of collagen I and III gene expression were observed, suggesting that cluster 13 represents modulated corneal endothelial cells, which have acquired a fibroblast phenotype; hence this population was assigned as fibroblastic corneal endothelial cells (FCECs). IF indicated a small number of the FCECs to be present in the stroma of limbal and peripheral cornea (Figure S11A, B).

Clusters 11, 18 and 20 displayed high expression of the atypical chemokine receptor (*ACKR1*, also known as DARC, Fig. 1B), whose expression is found on the endothelial cells of capillary and post-capillary venules [36]. Differential gene expression analysis (Figure S11C) identified *CDH19, CCL21* & *LYVE1* and *POSTN* to be predominantly expressed in clusters 20, 18 and 11 respectively. IF revealed the presence of CDH19 immunopositive cells in the corneal endothelium (Figure S11D-G) and POSTN immunopositive cells in the blood vessels (Figure S11H), leading us to define clusters 20 and 11 as corneal endothelium and blood vessels, respectively. Cluster 18 was defined as lymphatic vessels based on CCL21 and LYVE1 expression. IF indicated CCL21 immunopositive cells to be in the limbal and conjunctival region (Figure S11I).

#### Identification of red blood cells and melanocytes

Cluster 14 showed high expression Hemoglobin Subunit Alpha 1 (*HBA1*) and other genes present in red blood cells (Fig. 1B, S1): these were located under the conjunctival epithelium (Figure S12A) and the limbal crypts (Figure S12B). Cluster 19 showed a high expression of pigmented cell markers including *TYRP1, PMEL, MLANA, MITF* and *TYR* (Figs. 1B and S1), which led us to define this cluster as melanocytes. IF revealed the presence of MLANA and MITF double immunopositive cells under the limbal crypts (Fig. S12D). MLANA immunopositive cells were also found scattered in the limbal epithelium, corroborating previous findings of pigmented LSCs [37].

In summary, our scRNA-Seq data combined with IF analysis reveals the presence of progenitor and differentiated cells in all the layers of cornea and adjacent conjunctiva as well as the accessory cells. An interactive cell browser enabling users to view these data and perform interactive queries can be found at: http://retinalstemcellresearch.co.uk/CorneaCellAtlas/.

### scRNA-seq analysis reveals novel markers for LPCs

The scRNA-Seq analysis revealed an interesting LPC cluster in the limbal epithelium (cluster 9). To identify LPCs specific markers, we investigated which of the 119 highly expressed markers of cluster 9 (Table S1) was absent or expressed at low levels in the other corneal epithelial cell clusters. Five markers namely *GPHA2, CASP14, MMP10, MMP1* and AC093496.1 *(*Lnc-XPC-2) were highly and predominantly expressed in cluster 9 (Fig. 2A). IF showed strong and predominant GPHA2 expression in the limbal crypt overlapping with KRT15 expression (Fig. 2B–D, H, I), whilst MMP1 and MMP10 immunostaining was observed in the limbal (Fig. 2D, K, M) as well as in the corneal and/or conjunctival epithelium (Fig. 2J, L, O, P), hence the latter two markers cannot represent LPCs. A few *GPHA2* immunopositive cells within the limbal crypts, which were characterised by ΔNp63, p27 and CEBPδ expression (Fig. 2E–G), were also immunopositive for Ki67 (Fig. 2C, white arrow), indicating that most of GPHA2+ cells within the crypts are quiescent. Biomart bioconductor R package was used to annotate differentially expressed genes in cluster 9 with GO terms. GPHA2 (glycoprotein hormone subunit alpha 2) was annotated by GO to be located on the “cell surface”. Because of its predominant expression in the limbal crypts and cell surface location GPHA2 was selected for further investigations detailed in the rest of this section.

**Fig. 2:**
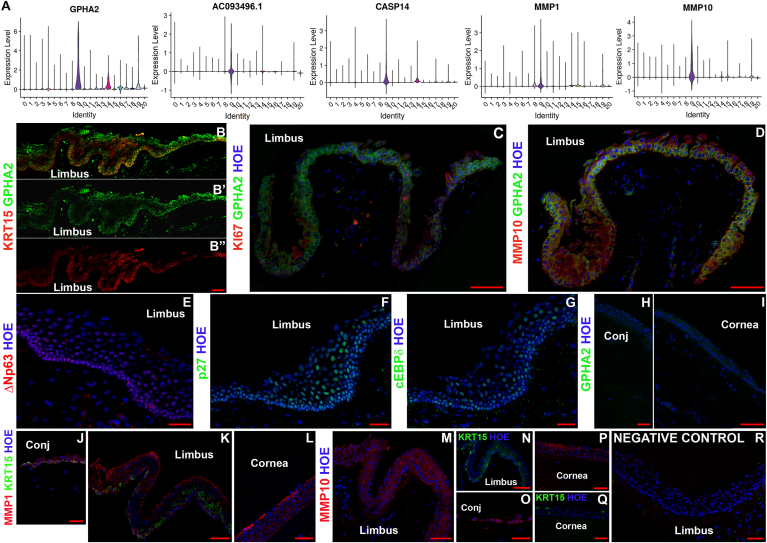
Novel markers for LPCs. A) Violin plots showing the expression of five transcripts (*GPHA2, CASP14, MMP1, MMP10 and* AC093496.1), which are highly and predominantly expressed in LPCs (cluster 9); B-D) IF analysis showing overlapping GPHA2 and KRT15 expression (B, B′, B′), rare co-localisation of GPHA2 with Ki67 expression shown by white arrow (C) and overlapping GPHA2 and MMP10 expression in the limbal crypts (D); E-G) IF showing expression of ΔNp63 (E), p27 (F) and CEBPδ (G) in the limbal crypts; H–I) faint background like-staining of GPHA2 in the conjunctival (H) and central cornea region (I); J-L) MMP1 is expressed in the conjunctival (J), limbal epithelium (K) and corneal epithelium(L); M − P), MMP10 expression is found in the limbal (M), conjunctival (O) and corneal epithelium (P); KRT15 expression in the limbal and corneal epithelium shown in N and Q panels represent sister sections to MMP10 expression in M and P panels; R) Representative negative control immunostaining. Representative images from four different human cornea and conjunctiva samples are shown. Scale bars: 50 μm. Nuclear staining indicated by Hoechst in blue colour.

Subsequently, we investigated the expression of GPHA2 on *ex vivo* expanded limbal epithelial cells (LECs). GPHA2 was present in a few cells clustered together in the middle of or periphery of colonies marked by KRT15 and ΔNp63 expression (Fig. 3A and B). All the GPHA2 cells were Ki67^+^; however not all Ki67^+^ cells were GPHA2^+^ (Fig. 3C). The same was also true for co-staining with ΔNp63, which was observed throughout the colonies with very few GPHA2+ΔNp63+ cells present (Fig. 3B) in a cluster like pattern. In accordance, flow cytometric analysis indicated 0.41% ± 0.21 (n = 7) of cultured LECs to display cell surface expression of GPHA2. In accordance with GPHA2's expression in LPCs, a significant reduction in expression was observed upon air-liquid interface induced differentiation of LECs (Fig. 3D). Flow activated cell sorting of GPHA2^+^ and GPHA2^-^ cells followed by qRT-PCR analysis indicated a significantly higher expression of *GPHA2* and *KRT15* in the GPHA2^+^ cells. Moreover, GPHA2^+^ cells were distinguished by their ability to generate holoclones at the expense of meroclones and paraclones (Fig. 3E).

**Fig. 3:**
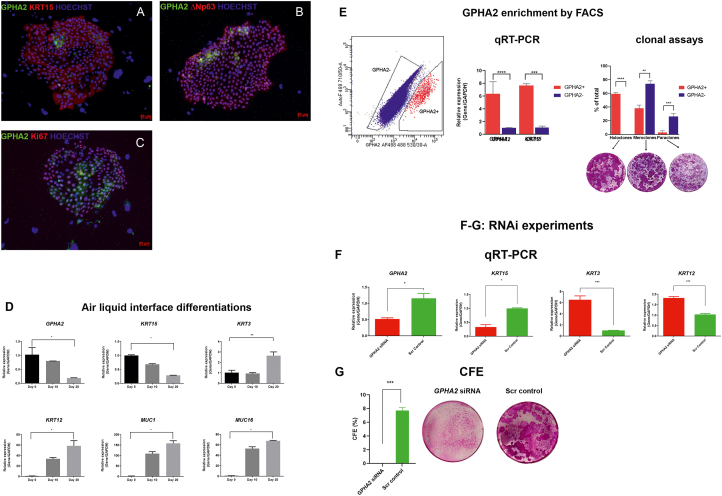
GPHA2 supports LPC undifferentiated state *in vitro*. A-C) Expression of GPHA2 and overlap with KRT15, ΔNp63 and Ki67 respectively in *ex vivo* expanded LECs, passage 1; D) Downregulation of *GPHA2* and KRT15 expression and upregulation of differentiated corneal epithelial markers, *KRT3, KRT12, MUC1* and *MUC16* upon air liquid induced differentiation of LECs; E) Flow activated cell sorting combined with qRT-PCR and clonal analyses indicate enrichment of cells with holoclone forming ability in the GPHA2+ enriched cell fraction; F) Quantitative RT-PCR analysis showing knockdown of *GPHA2* expression in *ex vivo* expanded LECs resulting in a significant decrease in *KRT15* and increase in *KRT3* and *KRT12* expression; G) CFE is significantly reduced upon GPHA2 knockdown . E–G: Data presented as mean ± SEM, n = 3 for panels E–F and n = 7 for panel G. Statistical significance was assessed using one-way Anova with Dunnet Multiple Comparison Tests, *p < .05; **p < .001, ***p < .001, ****p < .0001.

RNA interference (RNAi) was carried out to assess the role of GPHA2 in LECs clonogenecity and cell fate determination (Fig. 3F). Morphological and qRT-PCR analysis showed the presence of large, elongated cells in the *GPHA2* siRNA group (data not shown), indicative of differentiation onset, corroborated by a significant decrease in expression of *KRT15* and the increase in expression of the differentiation markers, *KRT3* and *KRT12* (Fig. 3F). Colony forming efficiency (CFE) assays indicated a huge reduction in the *GPHA2* siRNA group (Fig. 3G).

Taken together, this set of experiments suggests that GPHA2 positively controls the undifferentiated state of human LPCs, and that this new marker can be used to identify human LPCs.

### Combined scRNA-Seq and ATAC-Seq analysis reveals key transcriptional networks in LPCs and close interactions with immune cells

Four human adult cornea/conjunctiva samples were subjected to scATAC-Seq analysis. Approximately 10,000 cells were captured using the 10x Genomics Chromium Single Cell ATAC Library & Gel Bead Kit (version 1). Following QC, 10,625 cells were obtained after data integration. All the predicted RNA-Seq clusters were also found in ATAC-Seq analysis except for cluster 20, which may be due to the very low cell numbers present in this cluster (Figure S13). Interestingly, the LPCs cell cluster was distinct in the UMAP plot from the other epithelial clusters. To analyse these in more detail, 7618 epithelial cells were selected (Fig. 4A) and differential accessibility analysis of the LPCs (cluster 9, Table S3) versus the rest of the corneal and conjunctival epithelial cell clusters was performed. This analysis identified differentially accessible (DA) peaks overlapping with promoters (−1000bp, +100bp) and distal or intergenic regions of any transcription start site (Table S3). Amongst the top accessible promoters in LPCs were those of *KRT14* and *CASP14* (Fig. 4B and C), both highly expressed in this cluster (Figure S4C, 2A). Amongst the less accessible promoters in the LPCs cluster 9, we identified *MUC22* (Fig. 4D), which is highly expressed in the corneal and limbal superficial epithelial clusters (Fig. 1A), but not in LPCs.

**Fig. 4:**
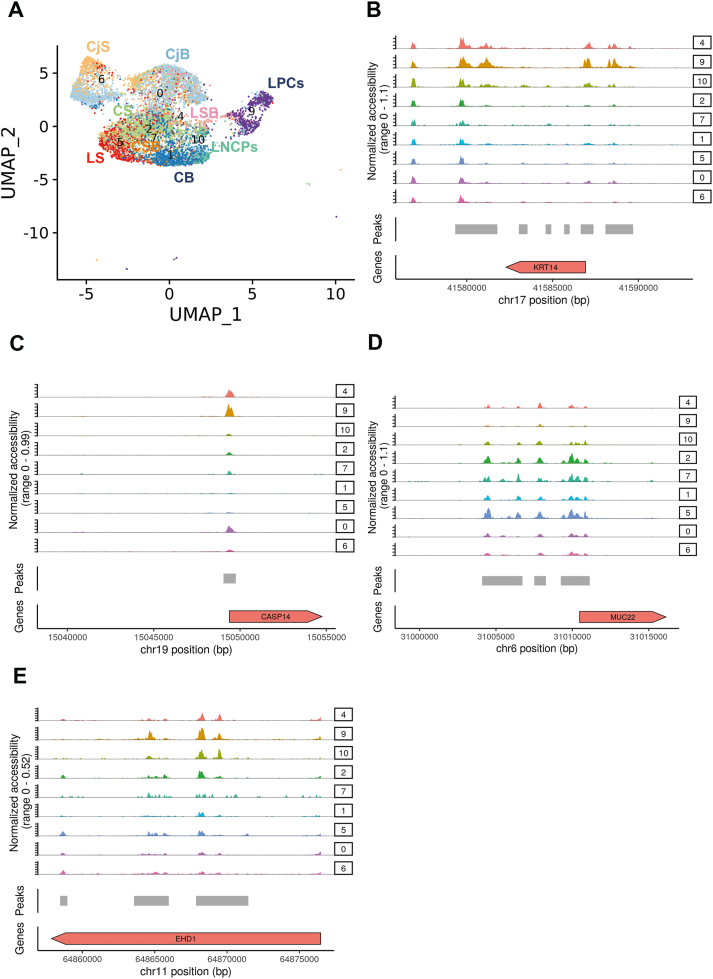
scATAC-Seq of adult human cornea and conjuctiva (see also Figure S13, Table S3). A) UMAP of adult human cornea and conjunctiva epithelial clusters and limbal neural crest progenitors; B-E) Schematic single cell chromatin accessibility of *KRT14* (B), *CASP14* (C), *MUC22* (D) and *EHD1* (containing a distal enhancer for *GPHA2*) (E) in the human cornea and conjunctiva epithelial clusters and LNCPs.

The JEME database [38] was used to link the differentially accessible peaks to the enhancer regions for LPCs (cluster 9) and limbal suprabasal cells (cluster 4). The *EDH1* locus, containing a distal enhancer of the LPCs marker, *GPHA2*, was identified as being more accessible in cluster 9 (Fig. 4E), in accordance with its putative function in maintaining LPCs undifferentiated state and self-renewal. Transcription factor (TF) binding motifs enrichment was performed using Signac (Table S3). It is of interest to note that binding factor motifs for the putative LPCs and progenitor markers, such as *TP63* and *CEBPD,* were enriched in the LPCs cluster (Table S3).

To identify TF networks governing the LPCs cluster the upstream regulator tool in IPA was used, combined with overlay analysis of DA peaks, enhancers, and TF binding motifs. This analysis revealed enhanced activation of 23 upstream regulators in LPCs compared to the rest of epithelial clusters (Table S4). Fourteen out of the 23 upstream regulators represent pro-inflammatory cytokines (TNF, IL1β, IL6, IL17A, IFNˠ and OSM), pro-inflammatory cell surface receptors (TREM1), inducers of proinflammatory cytokine expression (AP1) and regulators of inflammatory response (NFkβ, RELA, CSF2, PI3K, ERK1/2, and F2). Importantly, 6 of these regulators (TNF, IL1B, *IFNˠ, OSM, TREM1, CSF2*) show the highest expression in immune cell clusters 15 and 17 (Figure S14), whilst a further regulator (IL6) is predominantly expressed in immune cells and limbal fibroblasts (data not shown). Importantly, CellPhoneDB [39], revealed multiple significant interactions between LPCs (cluster 9) and immune cells (clusters 15 and 17, Table S5). To validate these findings, LECs were cultured in the presence of TNFα (10 ng/ml), IL6 (10 ng/ml) and IL1β (10 ng/ml) for 7 days with daily media changes. Cell counts indicated a significant decrease in cell number upon TNFα and IL1β treatment, corroborating published work implicating these two immune regulators in apoptosis of LECs [40] (Figure S15A). Quantitative RT-PCR and flow cytometric analysis indicated a significant increase in expression of GPHA2 and TP63 expression only in the TNFα treated group (Figure S15B). The increase in GPHA2 expression was also corroborated by flow cytometric analysis (Figure S15C). Together these data suggest that TNFα treatment group most likely induces the apoptosis of differentiated corneal epithelial cells, thus mimicking a central cornea defect [41], which in turn stimulates the proliferation of LPCs. This hypothesis is corroborated by clonal assays, which revealed a significant increase in the number of holoclones at the expense of meroclones in the TNFα treated group. A significant increase in paraclones was observed in IL1β treated LECs (Figure S15D). Together these findings suggest an important role for pro-inflammatory cytokines produced by the immune cells in the regulation of LPCs transcriptional profile, corroborating previous reports showing that supplementation of LECs cultures with pro-inflammatory factors can impact directly on the expression of putative LPCs markers and their colony-forming efficiency and size [28,40,42,43].

Amongst the upstream regulators, we also found several growth factors, namely EGF, BDNF and FGF2 (Table S4). Addition of EGF has become a standard media requisite for the expansion of LECs, with EGF addition stimulating proliferation [44], colony growth under serum free conditions [45] and inhibiting the expression of differentiation markers [46]. EGFR is present in LPCs [47] (Table S5) and its inhibition has been shown to affect epithelial cell proliferation during corneal wound healing [48]. Although a direct role for FGF2 in LPC expansion has not been proven, FGFR2 is essential for corneal epithelial cell proliferation and differentiation [49]. Similarly, the nerve growth factor BDNF, may influence LPCs, via promotion of corneal innervation [50]. One of the BDNF receptors, the low affinity nerve growth factor receptor, p75, is differentially expressed in LPCs and downregulated during LECs differentiation [51]. Impairment of trigeminal innervation, which provides trophic support to the cornea results in neurotrophic keratitis, a degenerative disease characterized by corneal sensitivity reduction, spontaneous epithelium breakdown, and impairment of corneal healing [52]. The presence of BDNF as an upstream regulator in LPCs may therefore underline the close interaction between LPCs and corneal nerves, contributing to the maintenance of corneal epithelial surface integrity and consequently ocular surface homeostasis.

It is interesting to note that pro and anti-angiogenic factors including Coagulation Factor 2 (Thrombin), VEGF and PDGF BB were found as upstream regulators of LPCs. While VEGF and PDGF BB are pro-angiogenic factors [53], thrombin itself can activate thrombospondins 1 and 2 (TSP-1 and TSP-2), which are expressed in the cornea and contribute to its avascularity [54]. Collectively, these data suggest a balanced action of pro-and anti-angiogenic regulators in LPCs to maintain an avascular state in the corneal epithelium.

#### LECs expansion *ex vivo* and limbal dysplasia *in vivo* result in downregulation of GPHA2 expression

*Ex vivo* expansion of LECs is widely used for treatment of patients with LSCD either by single cell disassociation of limbal rings and plating on mitotically inactivated 3T3 fibroblasts or explant outgrowth on a substrate such as human amniotic membrane (HAM) [55]. Although these techniques are widely used clinically, it is not known whether the *ex vivo* expanded LECs resemble the LPCs *in vivo* or if either technique is superior. To address these questions, we obtained one limbal ring from an 80-year-old living female (exenteration procedure), which we analysed by scRNA-Seq before and after *ex vivo* expansion on 3T3 feeders and HAM. 14,897 cells were obtained after filtering and QC steps. To facilitate cell cluster annotations, this dataset was integrated with the four adult human cornea/conjunctival datasets using integration methods developed by Stuart et al. [56], which are designed to overcome batch effects and identify shared cell identities across different experiments, whilst preserving the identity of cells that are unique to a specific cluster. We identified three additional clusters in the *ex vivo* expanded LECs (Fig. 5A and Table S6), all of which were characterised by a high percentage of cells in S and/or G2/M phase of the cell cycle (Fig. 5B) and high expression of *Ki67* and *PCNA*, indicative of their proliferative nature (Table S6).

**Fig. 5:**
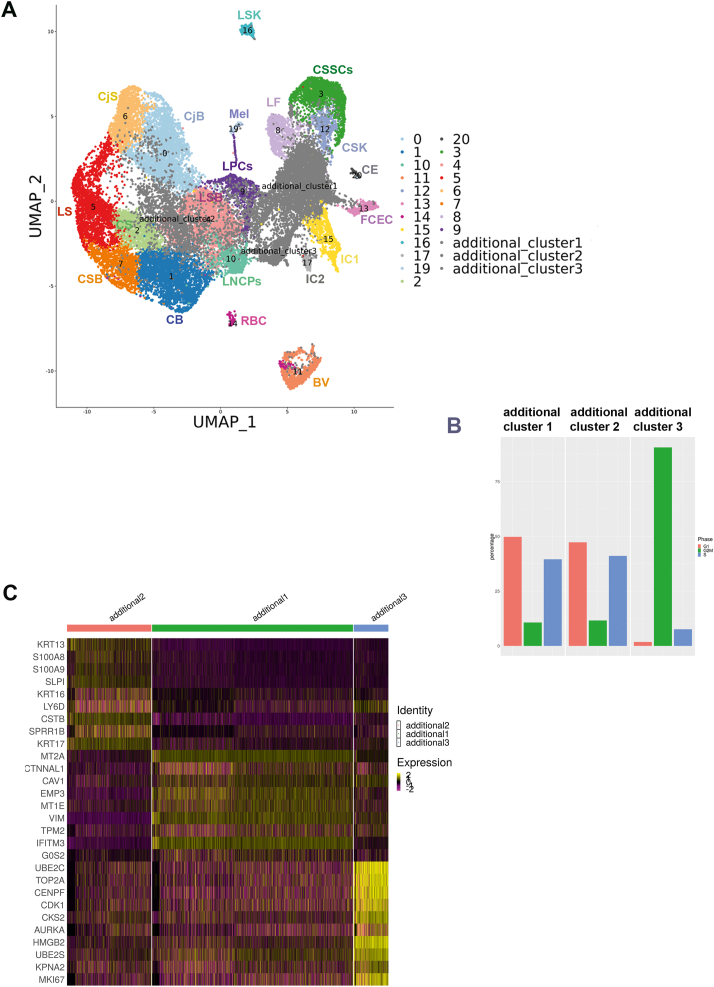
scRNA-Seq of *ex vivo* expanded human LECs and comparison to adult human cornea and conjunctiva (see also Figure S16 and Table S6). A) UMAP of *ex vivo* expanded LECs integrated with the four adult human cornea/conjunctiva samples shown in Fig. 1A, revealing the presence of three additional cell clusters found in the *ex vivo* expanded LECs. Although all cluster annotations are the same as in Fig. 1A, during the integration process the original 18 and 11 were combined in the integrated cluster 11; B) Cell cycle distribution of additional clusters 1–3; C) Comparative heatmap showing differentially expressed genes between the three additional clusters found in the *ex vivo* expanded LECs.

Differential gene expression analysis identified several markers associated with limbal basal epithelium, including *ITGA6* [57]*, ITGB1* and *VIM* [58] in the additional cluster 1, defining it as cultured basal limbal epithelium (Fig. 5C). Keratin 13 was the most highly expressed marker in the additional cluster 2 (Table S7). Published studies in the field have shown expression of KRT13 in the suprabasal limbal epithelium, for this reason we defined this cluster as cultured suprabasal limbal epithelium [59]. KRT13 is also a marker of conjunctival epithelium and we cannot fully dismiss that this cell cluster may represent a mixture of suprabasal limbal epithelium and cultured conjunctival epithelium, as limbal ring dissections can often include a small rim of surrounding conjunctiva. The additional cluster 3 was characterised by high expression of markers of mitosis in addition to epithelial progenitor markers *KRT14* and *KRT15* and was therefore annotated as mitotic epithelial progenitors. A correlation analysis was performed by taking the average gene expression of each cluster for the top 2000 highly variable genes used in the clustering analysis. This analysis indicated the additional cluster 1 to be more like the LPSs cluster 9 (correlation coefficient = 0.91), whilst the additional cluster 2 was similar to both limbal suprabasal cell cluster 4 and LPCs cluster 9 (correlation coefficient 0.93 and 0.92 respectively).

LECs culture on 3T3 feeders and HAM generated similar percentages of basal and suprabasal limbal epithelial cells and mitotic progenitors (Figure S16A). Differential gene expression analysis was used to compare *ex vivo* expanded cultured basal limbal cells (additional cluster 1) to LPCs *in vivo* (Figure S16B), revealing a significant decrease in the expression of LPCs markers (Table S7) including *GPHA2, TXNIP* and *CEBPD,* corroborating the presence of very few GPHA2 cells in *ex vivo* cultured LECs (Fig. 3A). In addition a significant upregulation of markers associated with highly proliferative basal epithelial cells (e.g. S100A2, *S100A10*) [19] was observed (Table S7 and Figure S16B).

To investigate if a similar phenomenon occurs during dysregulation of LPCs growth *in vivo*, we focused on scRNA sequencing of an adult cornea (deceased male 80 years old) with a visible limbal growth/dysplasia protruding from the limbus towards the nasal conjunctiva (Fig. 6A). The scRNA-Seq subset obtained from the cornea with limbal dysplasia was integrated with the adult cornea and conjunctiva and cultured LEC subsets as described above (Fig. 6B), resulting in the identification of six additional clusters (Table S8), five of which were present in both the cornea with limbal dysplasia and cultured LECs (Figure S17).

**Fig. 6:**
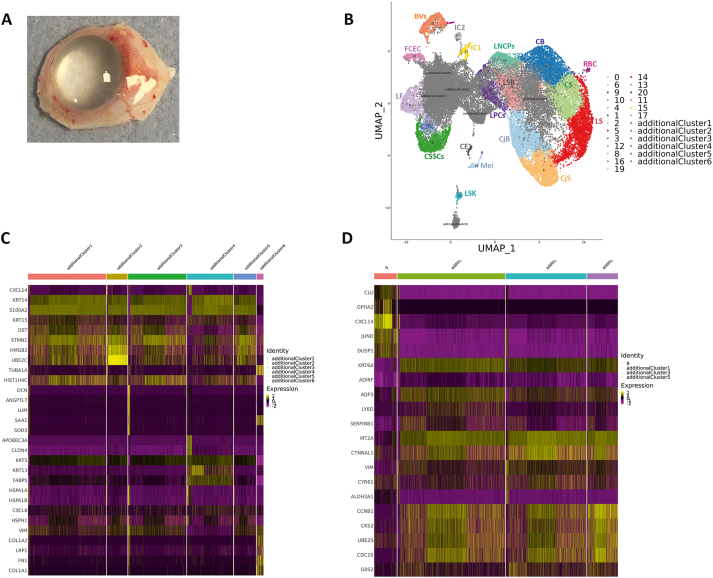
Expansion of LECs *in vivo* leads to loss of GPHA2 expression and acquisition of markers associated with proliferative limbal basal epithelial cells (see also Figures S17, S18 and Table S8). A) Representative photo showing a human cornea with a limbal dysplasia; B) UMAP of limbal dysplasia sample integrated with *ex vivo* expanded LECs (Fig. 5A) and the four adult human cornea/conjunctival samples (Fig. 1A) showing the presence of six additional clusters in the cornea with limbal dysplasia. Although all cluster annotations are the same as in Fig. 1A, during the integration process the original cluster 7 was combined with original cluster 2 in the integrated cluster 6 and clusters 18 and 11 were combined in the integrated cluster 11; C) Comparative heatmap showing the differentially expressed genes between the six additional clusters found in the cornea with limbal dysplasia; D) Comparative heatmap showing differentially expressed genes between LPCs (cluster 9) and the proliferative basal limbal epithelium I-III corresponding to additional clusters 1, 3, 5 in the cornea with limbal dysplasia.

The additional cluster 6, which is specific to the cornea sample with limbal dysplasia, displayed high expression of *SAA1*, a marker of pancreatic ductal adenocarcinoma tumor stroma primarily composed of cancer-associated fibroblasts [60] (Table S8). SAA1 is also expressed in the corneal fibroblasts and corneal keratocytes and shown to be upregulated in inflammation mediated neovascularization [61]. This cluster also displayed high expression of other fibroblast and keratocytes markers including *VIM, FN1*, *COLA1A2, TIMP2 (*Fig. 6C and Figure S18C*)* and for this reason was defined as activated fibroblastic stroma cluster.

The proliferation markers, *PCNA* and Ki67 were highly expressed in additional clusters 1–5, indicating their proliferative nature (Figure S18A). Cell Cycle analysis corroborated these findings (Figure S18B) showing a considerable percentage of cells in the S and/or G2/M phases of the cell cycle. Differential gene expression analysis indicated high expression of proliferative epithelial basal cell markers (*KRT14, KRT15,* S100A2 etc.) in the additional clusters 1–5 (Figure S18C). Additional cluster 4 showed high *KRT13* expression, a marker of suprabasal limbal epithelium [59] (Fig. 6C) and thus was defined as proliferative suprabasal limbal epithelium. Cluster 2 contained more than 95% of cells in mitosis and was defined as mitotic epithelial progenitors. The additional clusters 1,3 and 5 were defined as proliferative basal limbal epithelium I-III in view of their cell cycle characteristics and high expression of proliferative epithelial basal cell markers (*S100A2*) (Fig. 6C). Similarly, to *ex vivo* expanded LECs, the additional clusters 1, 3 and 5 showed a downregulation of *GPHA2* and *TXNIP* (Fig. 6D), indicating that limbal dysplasia *in vivo* also results in downregulation of some of the typical LPCs markers.

### scRNA-seq of keratoconus corneas reveals activation of collagenase in the corneal stroma and a reduced pool of limbal suprabasal cells

To validate the applications of single cell sequencing as a platform for gaining molecular insights into disease pathology we focused on keratoconus, an asymmetric, progressive disease in which the cornea becomes conical in shape. The aetiology of keratoconus is not fully understood although current knowledge postulates that this is a final common pathway for several diseases, which are underlined by genetic predisposition triggered by environmental factors [62]. To gain insights into disease pathology we performed scRNA-Seq of central cornea samples obtained from two keratoconus patients (18 and 43-year-old males) at the time of corneal transplantation and one cadaveric unaffected female adult subject (80 years old). After filtering and QC steps, 2641 cells were obtained. This dataset was integrated with the adult cornea/conjunctiva dataset using the same integration methods as above (Fig. 7A), resulting in identification of a new additional cluster of central stroma keratocytes (Table S9), which was present in the central cornea of both unaffected subject and keratoconus patients. Following the data integration, the percentage of cells in each cluster was compared between the two keratoconus patients and the central cornea unaffected subject (Fig. 7B), revealing a noticeable decrease in limbal suprabasal cells (cluster 4) and an increase in cluster 12 (corneal stroma keratocytes) in the keratoconus central cornea samples.

**Fig. 7:**
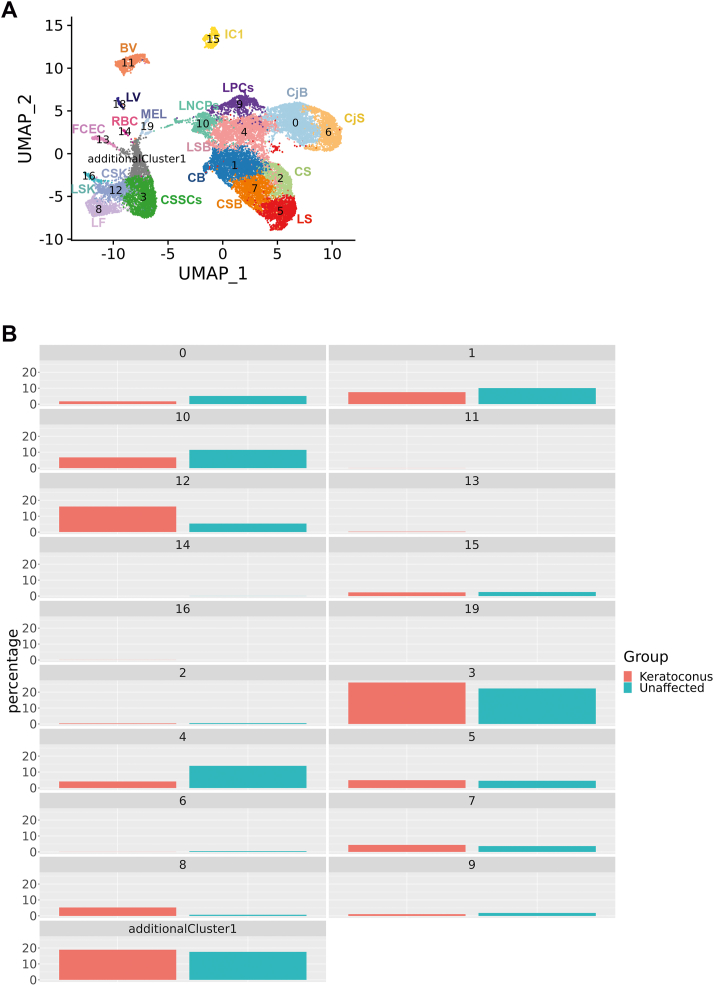
scRNA-Seq of keratoconus samples (see also Figures S19, S20 and Tables S9-S12). A) UMAP of central cornea samples obtained from two patients with keratoconus and one unaffected subject integrated with the four adult human cornea/conjunctiva samples shown in Fig. 1A. Although all cluster annotations are the same as in Fig. 1A, during the integration process the original, cluster 19 and cluster 20 matched just one cluster in the integrated clustering (shown as cluster 19), as did cluster 15 and 17 (shown as cluster 15); B) Comparative analysis between the unaffected and keratoconus cornea showing the percentage of cells in each cluster.

Irregular arrangement, enlargement and reduction in corneal basal epithelial cell density of keratoconus patients is known [63]. In addition, loss of corneal stromal architecture is a key feature of keratoconus affected corneas. In view of this and the changes noted in cluster 12, we performed differential gene expression analysis for this cluster between the unaffected subject and keratoconus patients (Figure S19A,B, Table S10), which indicated a significant downregulation of genes involved in collagen biogenesis (*COL8A1, COL6A3, COL5A2*), proteoglycans (*KERA*), genes involved in WNT signalling pathway (*WNT5A, DKK2, SFRP1*), serine protease inhibitors (*SERPINA5, SERPINE2*), mitochondrial genes (*MT-CO1,MT-ND1*), cytokeratins (*KRT5, KRT14*), cytokines (*IL32*) and members of the TGFβ (*TGFβI*) family known to be involved in collagen-cell interaction in keratoconus stroma samples, corroborating previously published data [[62], [63], [64], [65]]. In contrast, we noticed a significant upregulation of genes involved in ECM degradation (*TIMP3, TIMP2*), cell death (*ANXA1*), oxidative stress defence and detoxification (*ALDH3A1*), epithelial to mesenchymal transition (*VIM, TWIST1*) and FOS and JUN oncogenic transcription factors (*FOS, FOXB, JUNB, JUND*) (Fig. S19B).

The top canonical pathways that were most significant to keratoconus stroma were EIF2 and mTor signalling, oxidative phosphorylation, and mitochondrial dysfunction (Table S11). Interestingly these top pathways have been associated with keratoconus pathogenesis through proteomic studies of corneal stroma obtained from affected patients [[64], [65], [66], [67]], providing a validation benchmark for our single cell studies. Using IPA, we identified activated upstream regulators with the most significant for Keratoconus being collagenase (Table S12). The increase in collagenase activity, which has been biochemically confirmed in keratoconus stroma, is of great interest as it has been shown that this enzyme cleaves collagen molecules into fragments, which are processed by gelatinase (also activated in keratoconus stroma) and cathepsin for degradation, corroborating the observed decrease in collagen in the corneal stroma of keratoconus patients. Together our scRNA-Seq studies support the original hypothesis of Kao et al. that the decrease in collagen and stromal thinning in keratoconus is due to an increase in collagenase activity [68]; therefore keratoconus may represent a collagenolytic disease. The loss and degradation of stromal collagen may allow other cell types to repopulate the corneal stroma in keratoconus patients. Based on the significant increase in expression of *TWIST1*, a key transcription factor driving epithelial to mesenchymal transition and *VIM*, a key marker of mesenchymal cells, we speculate that the non-resident stromal repopulating cells may be of mesenchymal origin.

The typical presentation of keratoconus has both stromal and central epithelial thinning [63]; however the underlying cause for the epithelial thinning is not known. Given the decrease in percentage of cells observed in the limbal suprabasal cell cluster 4 in keratoconus patients (Fig. 7B), we performed a differential gene expression analysis for this cluster between the unaffected subject and keratoconus patients (Figure S20, Table S10), revealing a significant decrease in LPC *(KRT14, KRT15, TXNIP)* as well as a significant increase in expression of differentiated corneal epithelial cell markers (*AREG, KRT3, HES1*) in keratoconus samples. Together these data suggest that in keratoconus patients, the limbal suprabasal cells differentiate towards corneal suprabasal and/or superficial epithelial cells, depleting the pool of migratory limbal suprabasal cells that can repopulate the central corneal epithelium. Given previous interactions between immune and limbal suprabasal cells described earlier and the dominance of inflammatory driven signalling pathways in limbal suprabasal cells in keratoconus patients (Table S11), it may be possible that these changes are driven by inflammatory processes, also observed in tears of keratoconus patients [69].

### scRNA-seq of human developing cornea reveals stage-specific definitions of corneal epithelial, stromal, and endothelial layers

To understand the molecular events that lead to specification of stem and progenitor cells in the epithelial, stromal, and endothelial layers of the cornea, we performed scRNA-Seq analysis on seventeen human corneas dissected from karyotypically normal 10–21 post conception week (PCW) specimens. Following filtering and QC, 89,897 cells were analysed. High expression of typical epithelial, stromal and endothelial cell markers was not detectable at the very early stages of corneal development [70,71]; hence, we relied on published evidence of developmental tissue markers contributing to the corneal layer development (neural crest, periocular mesenchyme, mesoderm) and the transfer label function from Seurat to project cell annotations from the *adult cornea* into the developmental samples. All the scRNA-Seq data are deposited in the interactive cell browser http://retinalstemcellresearch.co.uk/CorneaCellAtlas/.

scRNA-Seq analysis indicated that at 10 PCW, a large cluster of neural crest cells was identified alongside a smaller cluster of mesodermal and proliferating progenitors (Fig. 8), showing high expression of *IGFBP5/PITX2/FOXC2* [72]*, PITX1* and *Ki67* respectively (Table S13). In accordance, IF revealed the presence of Ki67^+^ proliferating cells throughout the layers of developing cornea and conjunctiva (Figure S21A, B) as well as widespread expression of neural crest marker, FOXC2 (Figure S21C, D). At this stage of development, the limbus was distinguished by a thicker epithelium (2–3 cell layers) compared to corneal and conjunctival epithelium, which were mostly 1 cell layer thick (Figure S21E, F). The expression of putative LSCs/progenitor marker, ΔNp63 was observed throughout the basal epithelium of the ocular surface. KRT15 was expressed throughout the ocular surface epithelium in a patchy fashion with strong expression in the limbal area (Figure S21 E, F). Two epithelial cell clusters (cluster 2 and 3) were identified by the scRNA-Seq analysis and defined as corneal and conjunctival epithelium based on expression of *KRT3/12* and *KRT13/7* respectively (Fig. 8). The corneal epithelial cell cluster 2 was characterised by high expression of several cytokeratins (17, 18, 19, 8), mucins (15, 16, 20) and Metallothioneins (*MT2A, MT1X*) (Table S13). Scattered MT2A immunopositive cells could indeed be observed in the corneal, limbal and conjunctival epithelium (Figure S21G, H). Despite the expression of *KRT3* and *KRT12* transcripts in the corneal epithelial cell cluster 2, very few KRT3 and KRT12 immunopositive cells could be found by IF in the limbal region (Figure S21I, J). In contrast, strong expression of KRT13 was observed in the conjunctival and limbal region and few superficial cells of central cornea epithelium (Figure S21K, L), indicating an earlier specification of conjunctival epithelium and a peripheral to central wave of epithelial differentiation, as suggested earlier by Davies et al. [70]. The stromal cell clusters (keratocytes) were easily identified by the high expression of keratocyte markers, *TGFBI and THBS1* as well as the expression extracellular matrix components (*KERA, LUM,* various collagen chains) secreted by the corneal stromal keratocytes (Table S13) [73], which were also detectable by IF in the central cornea and limbal stroma (Figure S21M, N). The endothelial cluster was distinct from the others and expressed high levels of several genes found in developing endothelial cells including *KDR, MSX1, BMP2* and *COL4A2.*

**Fig. 8:**
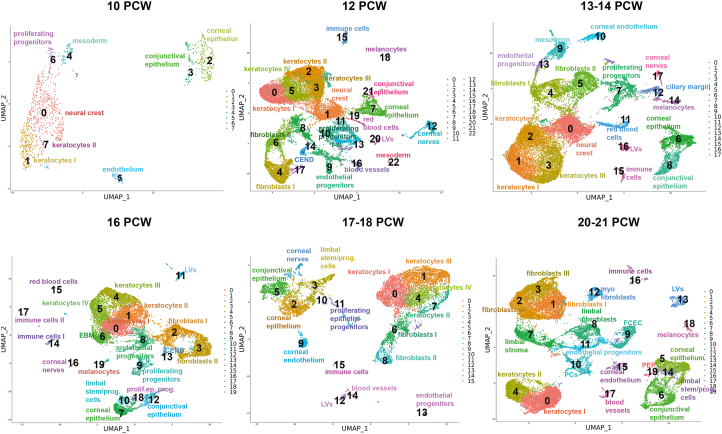
scRNA-Seq of embryonic and fetal cornea and conjunctiva from 10 to 21 PCW with cluster annotations (see also Table S13 and Figures S21-S26).

A much higher complexity was observed at 12 PCW, where the number of detected cell clusters reached 23 (Table S13). In addition to neural crest cell and mesodermal clusters, a higher complexity of corneal stromal populations was observed with increased cellularity (Fig. 8) alongside increased complexity of fibroblast cell clusters. The conjunctival epithelium (cluster 21) defined by *KRT13* and *KRT7* expression formed the smaller of the two epithelial clusters (Fig. 8). IF analysis revealed strong expression of KRT13 in the conjunctival and limbal epithelium and some superficial cells of the central cornea (Figure S22A). The corneal epithelium cluster 7 displayed high expression of *KRT15, KRT14, TP63* and *PAX6*, although KRT15 and ΔNp63 immunopositive cells were found throughout the ocular surface epithelium (Figure S22C, D), corroborating previously published data [70]. KRT12 immunopositive cells were observed in the limbal and corneal epithelium, whilst KRT3 was expressed in the superficial limbal epithelial cells (Figure S22E-G), indicating a more progressed stage of corneal epithelial differentiation and specification compared to 10 PCW. Three clusters of proliferating cells were also identified, indicative of ongoing cellular proliferation and corneal immaturity and consistent with Ki67 immunostaining throughout the corneal and conjunctiva (Figure S22A, B). scRNA-Seq analysis also revealed the presence of melanocytes, red blood cells, corneal nerves, and immune cells (Fig. 8, Table S13). At this stage of development, corneal endothelium and endothelial progenitor cells were present alongside two additional clusters representing the blood and lymphatic vessels (Fig. 8B). In accordance, CDH19 and TAGLN immunopositivity could be strongly observed in the developing corneal endothelium (Figure S22H).

The 13–14 PCW was very similar to 12 PCW in terms of cluster identity and the presence of accessory cells types (Fig. 8), although much less Ki67 immunoreactivity was observed (Figure S23A-C). At this stage of development, the corneal epithelium (cluster 6) defined by the expression of *KRT12* continued to show high expression of *CXCL14, KRT15, KRT14, KRT19, KRT15, TP63* and secreted WNT family members (*WNT6A, WNT10A, WNT4, WNT7B, WNT2B*) (Table S13). KRT13 was strongly expressed in the conjunctival and limbal epithelium and in few cells of the superficial corneal epithelium (Figure S23A-C), whilst ΔNp63 and KRT15 were expressed throughout the ocular surface epithelium (Figure S23D, E). KRT12 immunopositive cells were found in both limbal and corneal epithelium (Figure S23F, G), whilst KRT3 immunopositive cells were detected in the superficial limbal epithelium and in a few cells of the superficial corneal epithelium (Figure S23H, I).

Single cell RNA-Seq analysis of 16 PCW specimens indicated that the neural crest and mesodermal cell clusters were no longer detectable, suggesting their migration and differentiation to corneal stromal and endothelial cell layers was largely completed (Fig. 8). Two major differences were seen compared to 10–14 PCW. Firstly, the detection of a cell cluster (6) with high expression of Nidogen (Table S13), which may suggest the development of the corneal epithelial basement membrane, corroborating histological observations of a thin Bowman's layer in 15 PCW specimens [70]. Secondly, in addition to the well-defined corneal (cluster 7) and conjunctival epithelium (cluster 12), a cluster of proliferating epithelial progenitors (cluster 18) and limbal stem/progenitor cells (cluster 10) with high expression of *KRT15, KRT17, KRT19, PAX6* and *WNT4, WNT10A* and *WNT6* expression was present, suggesting the establishment of limbal and/or progenitor cells and corroborating previous scanning electron microscopy studies, revealing a clear demarcation of limbal epithelium containing CK15 immunopositive cells between the smooth cornea and conjunctiva at 16 and 17 PCW fetal specimens [70]. In accordance with scRNA-Seq findings, IF analysis revealed the presence of GPHA2 immunopositive cells in the limbal region and few cells of the corneal epithelium (Figure S24A, B) as well as the presence of limbal crypt-like structures with basal KRT15 and ΔNp63 expression and superficial KRT13 staining (Figure S24C-F). KRT3 was expressed in very few cells of the limbal epithelium with the majority of immunopositive cells localised to the corneal epithelium (as in the adult cornea), indicating a more advanced stage of corneal epithelium differentiation compared to 14 PCW (Figure S24G, H). KRT12 expression at this stage of development was confined to the corneal epithelium and few cells at the limbal region (Figure S24I, L).

The 17th and 18th week of development were characterised by very similar cell clusters to 16 PCW, however, there was increased cellularity of corneal and conjunctival epithelium compared to the other developmental stages (Fig. 8). The GPHA2 immunopositive cells were found in the limbal and corneal epithelium (Figure S25A-C), whilst KRT3 and KRT12 immunopositive cells were in the limbal and corneal epithelium (Figure S25D-G). KRT15 and ΔNp63 expression was observed throughout the ocular surface epithelium (Figure S25F-I); however, in contrast to the earlier development stages, KRT13 expression became restricted to the conjunctival and limbal epithelium (Figure S25J, K), indicating a better demarcation of the peripheral epithelium. Notwithstanding, the continuous expression of KRT15 throughout the ocular surface epithelium as well as GPHA2 in both limbal and corneal regions, suggests however that the boundary between the limbal and corneal epithelium has not been fully defined yet.

At 20–21 PCW, 4 epithelial clusters were detected comprising the corneal and conjunctival epithelium and proliferating epithelial progenitors and limbal stem/progenitor cells (Fig. 8). The immunohistochemical expression of key limbal stem and corneal and conjunctival epithelial markers remained much the same as in 18 PCW (Figure S26A-J) with strong GPHA2 expression in the limbal epithelium but less so in the corneal epithelium and KRT3/12 and KRT13 restricted to corneal and conjunctival epithelium respectively as well as the limbal epithelium. In accordance with the establishment of limbal stem/progenitor cells, clusters of limbal fibroblasts and stroma were identified (Fig. 8) and validated by IF (Figure S26K, L), suggesting the formation of a specialised limbal niche to support their self-renewal and differentiation. At this stage of development, an increase in fibroblast type and cellularity was observed. In addition, a defined cluster of myofibroblasts was identified (Fig. 8). The myofibroblast's key role is the restoration of corneal integrity because of their ability to secrete extracellular matrix, contribute to wound repair and adhesion capability to the surrounding substrates. In adults, development of corneal myofibroblasts is noticed after surgery and is considered as a pathological response to injury. Their presence at the midgestation stage may suggest a “beneficial” role in response to apoptosis that cornea is thought to undergo during morphogenesis, resulting in specification of the limbal ridge [70]. We were able to detect for the first time during corneal development, the FCECs cluster, which is characterised by the expression of markers involved in endothelial to mesenchymal transition, a process known to occur during *ex vivo* expansion of corneal endothelial cells or in response to inflammation. The identification of FCECs during midgestation may be linked to the extensive proliferation of endothelial progenitor cells during corneal morphogenesis.

In summary, our scRNA-Seq analysis of human developing corneas identifies stage-specific definitions of corneal epithelium, stromal and endothelial layers as well as the accessory cell types involved in maintenance of the limbal progenitor cells.

## Discussion

Advances in single cell sequencing technologies have enabled detailed studies of tissues and organs during human development and in adulthood [74]. We report here a comprehensive scRNA-Seq analysis of human cornea and conjunctiva encompassing 10–21 PCW and adult samples, providing a detailed map of corneal layer development and cell differentiation. We have used this analysis to identify new markers for LPCs *in vivo*, characterise the changes they undergo during cellular expansion and uncover the transcriptional networks and upstream regulators that maintain LPCs potency. Deposition of our data in open access databases and provision of an interactive cell browser provides a unique opportunity for other researchers to expand this analysis and get new insights on all corneal and conjunctival cell populations and their development.

During development, the eye is constructed from three sources of embryonic precursors: neuroectoderm, surface ectoderm and periocular mesenchyme [72]. The periocular mesenchyme receives cells from both the neural crest and mesoderm and contributes to multiple mature cell lineages including the corneal endothelium and stroma. Accordingly, our scRNA-Seq showed a large presence of neural crest and mesoderm cells at 10 PCW: these decreased significantly at 12 PCW, coinciding with the expansion of the stromal compartment (keratocytes) and the emergence of blood vessels, melanocytes, and corneal nerves. The corneal epithelium itself is formed from bilateral interactions between the neural ectoderm-derived optic vesicles and the cranial ectoderm [12]. Morphological and ultrastructural studies have shown a segregation between epithelial and stromal cells as early as 6.5 PCW; however, a detailed understanding of conjunctival, corneal and limbal epithelium specification until now is lacking. Our combined scRNA-Seq and IF analyses suggest a peripheral to central mode of differentiation with conjuctival epithelium being specified first, followed by gradual definition of corneal epithelium between 12 and 16 PCW and the establishment of limbal stem/progenitor cells at 16 PCW. The later establishment of limbal stem/progenitor cells could be due to the lack of limbal niche, which is essential for their maintenance. Although some elements of the niche (e.g. corneal nerves, melanocytes, blood vessels) are present from 12 PCW, the limbal stroma and fibroblasts, that are essential for the generation of necessary ECM and paracrine factor secretion, only become visible at midgestation (20–21 PCW).

Similarly, the corneal endothelium and blood and lymphatic vessels are present as early 12 PCW, with a source of endothelial progenitors established at 16 PCW. Although the endothelial progenitors were present until midgestation, we were unable to locate a similar cluster in adult cornea, which coincides with the inability of corneal endothelium to regenerate [75]. It is of interest to note the presence of a different population in adult cornea, identified as FCECs, which are thought to participate in endothelial wound healing under pathological conditions. The FCECs are typical not only of the adult stage, as our single cell analysis also uncovered their presence at midgestation (20–21 PCW) alongside the endothelial progenitor cell cluster. Together these data suggest the establishment of a “reserve endothelial” population at midgestation that may contribute to endothelial wound healing once the endothelial progenitors are no longer present (as in the adult cornea).

The corneal stroma keratocytes expand from 10 to 18 PCW before consolidating into two clusters at midgestation stages. Their development is associated with high expression of key stroma markers including lumican, keratocan and various collagen chains, necessary for the structural integrity of the corneal stroma. The presence of multiple keratocyte clusters can be explained by various states of maturation and proliferation in this compartment as well as their location either at the center or periphery of the cornea. Future studies employing high-resolution spatial transcriptomics-based techniques, should enable detailed localisation of these clusters and their developmental maturation. Literature precedence suggests the presence of CSSCs, which are believed to be of neural crest lineage and able to divide extensively *in vitro* and to generate adult keratocytes [11]. We were able to identify proliferating progenitors throughout corneal development, however the high expression of cell cycle markers did mask any lineage markers, for this reason we are unsure whether they comprise the CSSCs. Nonetheless, we can detect CSSCs in the adult cornea as a separate cluster associated with high expression of matrix metalloproteinase MMP3 and CD105. There have been extensive discussions in the literature whether CSSCs are bone marrow or neural crest derived. We were unable to observe expression of neural crest markers (*MITF, PAX6, SOX9* etc.) in the CSSCs cluster; however, our analysis did uncover a population of neural crest progenitors (LNCPs) located next to limbal basal epithelium, expressing neural crest markers (*PAX6, MITF*) and appreciable levels of *Ki67*. Together these findings suggest that CSSCs and LNCPs are two different cell populations, with the first most likely to contribute to the stroma regeneration and the second to the maintenance of LSCs.

The renewal of corneal epithelium is critically reliant on limbal stem and progenitor cells. Loss of LSCs results in limbal stem cell deficiency (LSCD) characterised by corneal neovascularization, chronic inflammation stromal scarring, corneal opacity and loss of vision [76]. Transplantation of autologous *ex vivo* expanded limbal stem and progenitor cells from a healthy contralateral eye onto the patient's damaged eye is an established treatment for patients with total/severe unilateral LSCD [7,77]. The frequency of stem and progenitor cells in the transplanted cell population is a key success factor for restoration of vision [78]; hence identification, quantitation and enrichment of limbal stem and progenitor cells prior to transplantation is an important and yet unmet area for research. Several putative limbal stem and progenitor cells markers have been identified including ΔNp63, ABCG2, ABCB5, C/EBPσ, Bmi1, Notch-1 amongst others [79,80]. To identify novel LPCs markers, we took an unbiased approach by identifying highly expressed marker genes found in LPCs relative to other epithelial clusters. We found five new genes not previously associated with LPCs, namely *GPHA2, CASP14, MMP10, MMP1* and AC093496.1 *(*Lnc-XPC-2). Two of these markers, namely MMP10 and MMP1 were also expressed in the conjunctival and central corneal epithelium and thus cannot represent LPC markers, whilst GPHA2, a cell surface marker was predominantly expressed in the limbal crypts, overlapping in expression with KRT15 and ΔNp63. A minority of GPHA2+ expressed Ki67, indicating that the majority of the GPHA2 immunopositive cells in the crypt are quiescent. Our IF analysis also showed a strong and abundant limbal crypt specific expression of GPHA2, which resonates well with recent finding in the field, indicating that stem/progenitor cells in the mouse corneal and skin epithelium as well as esophagus and gut epithelium are abundant and follow stochastic rules and neutral drift dynamics, dictated by neutral competition between clones and the tight spatial boundary of the stem cell niche [[81], [82], [83], [84]]. Downregulation of *GPHA2* using RNAi significantly reduced colony forming efficiency and induced cell differentiation, indicating an important role for GPHA2 in maintaining undifferentiated state of human LPCs. The enrichment of GPHA2+ cells by flow activated cell sorting combined with clonal assays, indicated a significant enrichment in holoclones, further supporting the data obtained from RNAi studies and validating GPHA2 as a cell surface marker for LPCs. Importantly, GPHA2 was expressed in the limbal epithelium from 16 PCW of human development, a time window, which corresponds with the first detection of LSCs/progenitor cells by scRNA-Seq analysis. It is of interest to note that a recent pre-print describing scRNA-Seq and clonal dynamics in mouse cornea, has also identified Gpha2 as a marker of quiescent LSCs [84], with similar expression pattern in the limbal crypt. GPHA2 is a glycoprotein hormone, which together with a second glycoprotein hormone β5 (GPHB5) forms the corticotroph-derived glycoprotein (CGH). Transgenic mice overexpression GPHA2 or GPHB5 have been generated and studied mostly in the context of thyroid function and morphology [85]. Whilst, GPHA2 transgenic mice do not show overt abnormalities, the GPHB5 developed proptosis or bulging eyes, which can be a consequence of trauma or swelling of surrounding tissue and may be of interest to follow up in the context of potential role for these glycoprotein hormones in limbal stem and progenitor cell biology and wound healing.

Given the importance of *ex vivo* LECs expansion for LSCD treatment, we used scRNA-Seq to compare cells before and after *ex vivo* expansion under two different culture conditions: 3T3 feeders and HAM. Although no significant differences were observed between the two expansion methods, the *ex vivo* LECs showed a significant downregulation of the LPCs marker identified in this study (*GPHA2*) and acquisition of markers associated with proliferative basal limbal epithelial cells. The same phenomenon was observed in limbal dysplasia *in vivo*, suggesting that this change in transcriptional profile could be due to several events including stimulation of proliferation as well as the incomplete replication of limbal niche under *ex vivo* culture conditions.

Our integrated single cell analysis revealed very close and well-balanced interactions between LPCs and progenitor cells with the immune cells, blood cells and corneal nerves. The interactions between the LPCs with immune cells were mimicked under *in vitro* culture conditions by supplementation of culture media with low concentration of inflammatory cytokines for a short window of time, one of which (TNFα) resulted in an increase in GPHA2 expression and holoclone forming cells. The limitation of this approach rests with the simplified nature of the experiment where single cytokines are added, which does not consider the more complex cytokine milieu of human limbus *in vivo* either in steady state conditions or increased inflammation such as in the case of LSCD. Despite this, the positive interactions between LPCs and inflammatory marker expression resonate closely with the recent findings from Altschuler et al. indicating loss of LSCs quiescence and delayed wound healing in mice lacking T cells [84]. Together these data suggest the need for a complete replication of the limbal niche during *ex vivo* propagation methods to fully support LPCs survival, self-renewal, and potency before clinical cell-based interventions. An interesting observation is that *ex vivo* expanded LECs with the current methods (HAM or 3T3 feeders) confer an overall 75% clinical success rate in cultured LECs transplantation. This suggests that *ex vivo* expanded LECs may regain their gene expression profile akin to LPCs upon re-establishing interactions with the limbal niche *in vivo*.

Finally, we were also interested to validate the application of single cell sequencing as a platform for gaining insights into corneal diseases. We focused on keratoconus, a corneal disorder characterized by progressive thinning and changes in the shape of the cornea, which affects approximately 1 in 2000 individuals worldwide. To date there have been several proteomic studies of corneal stroma from affected patients [[64], [65], [66], [67]], revealing EIF2 and mTor signalling, oxidative phosphorylation, and mitochondrial dysfunction to be at the heart of molecular changes in these patients. These same pathways were identified from the scRNA-Seq of only two patients and one unaffected subject. These findings when validated in a larger number of patients, may have important implications for future treatment of these patients.

In summary, the single cell analyses described herein provides a comprehensive cell type specific information for all the cells and layers found in the adult human cornea, limbus, and surrounding conjunctiva. By expanding this analysis for the first time to the developmental cornea and conjunctiva samples obtained from 10 to 21 PCW, we were able to determine the stage specific definitions of conjunctival, corneal and progenitor epithelial cells populations, establishment of the limbal niche as well as segregation of stroma and endothelium and their associated progenitor cell subpopulations. Importantly we identified a new putative LPCs surface marker, GPHA2 with important function in maintaining undifferentiated state of human LPCs. Our results also provide an excellent platform for using scRNA-Seq to compare the *ex vivo* expanded LECs to their native counterparts and comparing two different widely used cell expansion methods. Analysis of these datasets indicated that culture methods do not account for the transcriptional differences observed between the expanded limbal epithelial cells and LPCs *in vivo*. The very close interactions between LPCs and the different elements of the niche (immune cells, blood cells, corneal nerves, limbal fibroblasts and stroma) bring to the forefront the importance of limbal niche in maintaining the LPCs potency. Overall, the data presented herein, showcase the ability of scRNA- and ATAC-Seq to assess multiple datasets from the developmental and adult cornea, particularly the limbus under normal and disease states in a comprehensive manner, revealing genes/pathways that could lead to improvement in *ex vivo* expansion methods for cell-based replacement therapies for corneal disease and repair of the limbal niche before or as part of clinical interventions.

## Materials and methods

### Human tissue donation

Adult human eyes and corneal-scleral rings were donated for research following informed consent. All tissue was provided by NHS Blood and Transplant Tissue and Eye Services or the Newcastle and Sunderland NHS Trust following ethical approval (18/YH/04/20). Human tissue was handled according to the tenets of the Declaration of Helsinki and informed consent was obtained for research use of all human tissue from the next of kin of all deceased donors, or patient themselves, who were undergoing exenteration procedures.

Corneas from adults aged 51, 74, 77, 83 and 86 years old were used for the scRNA- and ATAC- analyses. Cell cluster validation by immunofluorescence (IF) was performed on four additional corneas obtained from adult donors aged 80, 82, 88 and 88 years old. Flow activated cell sorting, air liquid interface differentiations and RNAi experiments were performed on *ex vivo* expanded LECs from three different donors aged 78- and 80-years old undergoing exenteration procedures. Similarly, comparison of *ex vivo* expanded LECs on 3T3 feeders and HAM to LPCs *in vivo* was performed using a cornea obtained from an 80-year-old female, following an exenteration procedure. The cornea with limbal dysplasia was obtained from a deceased 80-year-old male, whilst corneas used for the keratoconus study were from 18 to 43-year-old males; these were compared to a cornea of an 80-year-old unaffected female subject. The inflammatory cytokine study was performed on *ex vivo* expanded LECs obtained from two 80-year-old and one 78-year-old donor, who had undergone exenteration procedures.

The human fetal material was provided by the Joint MRC/Wellcome Trust (MR/R006237/1) Human Developmental Biology Resource [86] under ethics permission 08/H0906/21 + 5 issued by the North East-Newcastle and North Tyneside 1 Research Ethics Committee. Seventeen samples obtained from 10 to 21 PCW terminations with no apparent abnormalities were used for the scRNA-Seq. Representative corneal specimens were selected for IF analysis: this included triplicate specimens from 10, 12, 14, 16, 18 and 21 PCW.

### Single cell sequencing

Cornea-conjunctival tissue was excised from donated eyes and dissociated to single cells using a multi tissue dissociation kit (Miltenyi Biotec). Dissociation time varied from 15 to 45 min depending on the age of the tissue. The gentleMACS Dissociator (Miltenyi Biotec) was used to aid dissociation of the adult tissue. Cell count and viability were monitored using a Tali Image-Based Cytometer and Viability Kit (Thermo Fisher Scientific).

For scRNA-Seq cells were captured and libraries generated using the Chromium Single Cell 3′ Library & Gel Bead Kit, version 3 (10x Genomics). scRNA-Seq libraries were sequenced to 50,000 reads per cell on an Illumina NovaSeq 6000.

For scATAC-Seq a nuclei preparation from the dissociated cells was performed following recommendations from 10x Genomics. The subsequent nuclei were captured, and sequencing libraries generated using the Chromium Single Cell ATAC Library & Gel Bead Kit, version 1 (10x Genomics). scATAC-Seq libraries were sequenced to 25,000 reads per nucleus on an Illumina NovaSeq 6000.

### Analysis of single cell RNA sequencing

#### Quality control

The sequenced samples were de-multiplexed and aligned to human reference genome GRCh38 before being quantified using CellRanger version 3.01. Quality control filtering was applied to remove any cells where fewer than 1000 reads or 500 genes or greater than 15% mitochondrial reads. Diagnostic plots were used to determine the most appropriate thresholds for our data. DoubletFinder was used to predict doublets in the data, which were then filtered.

#### Normalisation and batch correction

The Seurat R package (version 3.1.3) was used to normalise individual experiments using the “LogNormalize” method. The Seurat standard integrated analysis approach was used to overcome batch effects and combine samples from the developmental and adult samples [56]. Firstly, we selected a subset of 2000 genes, which were highly variable using the “FindVariableFeatures”. We then chose the first 30 principle components, which were used for integration. A combined dataset was created by finding anchors between the individual datasets to create a batch corrected expression matrix.

#### Cluster analysis and differential expression analysis

The corrected datasets were then clustered using a graph-based clustering. We used Clustree to assess the stability of clusters from a resolution of 0.2–1 and determined that a resolution 0.6 gave the highest number of stable clusters with cells from each donor represented in each cluster. The FindMarkers function identified markers for each cluster. Cell types were then assigned to these clusters and annotated using these genes lists. Cluster identity was validated using IF. The clustering results were visualised using uniform manifold approximation and projection (UMAP).

#### Label transfer and integration of datasets with adult samples

Integrated cluster analysis, described above, was performed on the following groups of developmental samples: 10PCW, 12PCW, 13-14PCW, 16PCW, 17-18PCW, 20–21 PCW. The adult dataset was used as a reference to predict cell identities in the developmental data using the Seurat “TransferData” function.

To compare the patient samples and LPCs with the adult cells we chose to integrate the datasets directly using methods developed by Stuart et al. [56] this was done to compare cells in non-overlapping populations to understand the cellular composition of the samples. This method allows comparison of cells within a shared space, as well as cells, which are unique to a specific tissue. We performed cluster analysis on the integrated datasets and then matched the original cluster labels from the adult data to clusters obtained within the integrated datasets. We then ran the dimension reduction steps on the integrated datasets to produce the UMAP. To aid interpretability we used the same colour scheme as the adult dataset.

#### Pseudotime trajectory analysis

Cells within the epithelial clusters 1, 2, 4, 5, 7 and 9 from the adult datasets were selected for pseudotime analysis. The Harmony batch correction method, which uses soft clustering to overcome over-discretisation was used to remove batch effects between donors. A trajectory and ordering of cells were inferred using Monocle 3.

#### Network analysis

The gene lists were analysed using the upstream regulator function from QIAGEN Ingenuity Pathway Analysis (QIAGEN IPA). CellPhoneDB was used to predict interacting cells across clusters. The analysis was performed using the clusters identified by Seurat.

## Analysis of single cell ATAC sequencing

### Peak detection and selection

CellRanger ATAC (version 1.2) was used to identify accessible regions from scATAC-Seq sequencing files. The cellranger-atac agrr module was used to create a set of shared peaks present across all four samples.

#### Quality control

The samples were then analysed with Signac version 0.2.4 along with Seurat. The percentage of reads in peaks, number of reads in percentage of reads in peaks per cell, percentage mapping to ENCODE blacklist, nucleosome signal and TSS enrichment was calculated. The data was filtered using thresholds to remove cells where the number of fragments within peaks was less than 1000 or greater than 50000, or with a blacklist ration of greater than 5% or a nucleosome signal of greater than 10 and a TTS enrichment of greater than 2.

#### Normalisation, batch correction and label transfer

Term frequency-inverse document frequency (TF-IDF) normalisation was applied to the data. 10000 peaks were chosen, which were close to the highly variable genes detected in scRNA-Seq data. Singular value decomposition (SVD) was then run on the TD-IDF matrix, using the selected peaks. UMAP clustering was used to visualise the data in 2D. A gene activity matrix was calculated in the scATAC-Seq dataset, using the closest genes to the peaks. Seurat was then used to perform clustering analysis on each sample and transfer the cell type labels from the scRNA-Seq to the scATAC-Seq data. Seurat integration methods were applied to the data peak accessibility to overcome donor effects and combine the four scATAC-Seq datasets.

#### Differential accessibility, classification of peaks and motif analysis

Logistic regression (LR) was used to identify differential accessible peaks between the different cell types. Peaks were linked to promoters using annotation from the Cellranger ATAC pipeline. The JEME database (https://www.nature.com/articles/ng.3950) was used to predict peaks in enhancer regions and the JASPAR 2020 database was used to look for the presence of motifs in differentially accessible peaks. Signac was used to create “pseudo-bulk” accessibility tracks by grouping cells by cell type within promoter/enhancer regions of key genes.

#### Adult and developmental human cornea and conjunctiva IF

The adult and developmental eyes were fixed in 4% PFA overnight followed by three washes in phosphate-buffered saline (PBS), incubated overnight in 30% sucrose/PBS, embedded in optimum cutting temperature (OCT) embedding matrix (Cellpath) and frozen at −20 °C. 10 μm cryostat sections were collected using a Leica Cm1860 cryostat (Leica). Cryosections were air-dried, washed several times in PBS and incubated in blocking solution (10% normal goat serum, 0.3% Triton-X-100 in PBS) for 1 h at room temperature. Slides were incubated with the appropriate primary antibody overnight at 4 °C (Table S14). After rinsing with PBS, sections were incubated with the secondary antibody for 2 h at RT. Alexa Fluor 488 and 546 secondary antibodies (Invitrogen-Molecular Probes) were used at a 1:1000 dilution. Negative controls were carried out by omitting the primary antibody. Afterwards, sections were washed three times in PBS and mounted with Vectashield (Vector Laboratories) containing 10 μg/ml Hoechst 33342 (Life Technologies) for counterstaining nuclei. All details of antibody purchase and concentrations are provided in Table S14.

#### Human limbal explant culture

Adult human limbal tissue was obtained from the surplus cornea-scleral rings remaining from cornea-scleral buttons provided by NHSBT for full thickness corneal grafts (penetrating keratoplasty) procedures, or exenteration procedures performed by NuTH Hospitals NHS Foundation Trust. The human amniotic membrane (HAM) was obtained from placentas donated during elective caesarean section deliveries and supplied by NHSBT. HAM is processed, mounted on nitrocellulose paper and frozen at −80 °C in 50% glycerol/Hanks solution and supplied as individual 3 cm^2^ units.

LEC culture was performed by preparing the epithelial medium, HAM construct and limbal biopsy preparation and placement onto the HAM. Epithelial medium was prepared to the following composition: 66.75% Low Glucose Dulbecco's Modified Eagle Medium (DMEM), 22.25% Ham's F12 medium (Gibco), Human Serum AB 10%, hydrocortisone 0.4 μg/ml, insulin 5 μg/ml, triiodothyronine 1.4 ng/nl, adenine 24 μg/ml, cholera toxin 8.4 ng/ml and EGF 10 ng/ml (Sigma Aldrich), then sterile filtered via 0.2μ filter (ThermoFisher Scientific). Three separate aliquots of the resultant media were further supplemented with 1% penicillin/streptomycin (Gibco). The HAM was defrosted at room temperature and washed twice by submersing the tissue in Dulbecco's Phosphate Buffered Saline (DPBS), (Gibco), supplemented with 1% penicillin/streptomycin. Subsequently, the tissue was washed a third time in 1% penicillin/streptomycin supplemented epithelial medium. Two 24 mm^2^ glass coverslips were prepared as per the HAM washing procedure, in separate wells to the HAM. One glass coverslip was carefully placed onto the lid of a sterile 6 well plate using sterile forceps and a drop of 1% penicillin/streptomycin supplemented epithelial medium added.

The HAM was carefully separated from the nitrocellulose paper with sterile forceps and stretched over the coverslip, avoiding air bubbles, and ensuring the epithelial side of the HAM was facing up, verified by brief application of a cellulose eye spear to the HAM. The overhanging edges of the HAM were trimmed using a sterile scalpel leaving approximately 1 mm overlap on each side. The second glass coverslip was placed on the edge of the 6 well plate lid and a drop of media added. The HAM/coverslip was carefully lifted using the edge of a sterile scalpel blade and sterile forceps then the edges were tucked under the coverslip. The HAM was secured by placement on top of the second coverslip, avoiding air bubbles between the coverslips and ensuring that the HAM remains flat and *in situ* throughout the culture process. The HAM construct was placed into a well of a fresh sterile 6 well plate with a drop of media underneath the construct, preventing air bubbles. Finally, the HAM was covered in the epithelial culture medium supplemented with 1% penicillin/streptomycin.

The limbal biopsies were prepared from the human cornea-scleral rings, dissected to 2 mm^2^ consisting of approximately 1 mm^2^ peripheral cornea and 1 mm^2^ adjacent cornea, ensuring all the cornea-scleral limbus was included. Due to the manual dissection, small rims of surrounding conjunctiva were also present in the dissected limbal rings. The stromal side remained in place. Each biopsy was placed at the centre of the prepared HAM, the spent epithelial medium removed prior to biopsy attachment. After an interlude of 2 min, 1% penicillin/streptomycin supplemented epithelial medium with was added slowly to each culture to ensure the explant was covered in medium without causing it to detach from the HAM. The cultures were fed with antibiotic-free epithelial medium on the third day and then every other day thereafter. Cultures were terminated when outgrowth reached approximately 90% confluence over the HAM. All cultures were performed under identical conditions and incubated in a tissue culture incubator at 37^O^C humidified with 5% CO_2._

### Human limbal epithelial cell culture on 3T3-J2 feeder layers

Adult human limbal tissue was obtained from the surplus cornea-scleral rings with informed consent as described above.

Twenty-four hours before LEC isolation from cornea-scleral tissue, mitotically inactivated 3T3-J2 (Karafast, USA) mouse fibroblasts were suspended in high-glucose DMEM supplemented with bovine calf serum (10%) (Hyclone, USA) and penicillin/streptomycin (1%) (Thermo Fisher Scientific, USA) and plated in a 9.6 cm^2^ tissue culture well at the final density of 2.4 × 10^4^ cells per cm^2^ as previously described [87]. The 3T3 cell suspension was placed in a tissue culture incubator at 37 °C overnight to allow the establishment of a 3T3 feeder layer. On the following day, LECs were harvested from cadaveric cornea-scleral rims as previously described [88]. The deeper layers of the cornea-scleral rings were dissected away together with excess sclera leaving a ring containing approximately 2 mm of peripheral cornea and 2 mm of adjacent conjunctiva. The remaining tissue containing limbal epithelium was then cut into smaller 1 mm^2^ pieces. The LECs were isolated from these pieces using serial trypsinization with 0.05% trypsin-EDTA solution (Thermo Fisher Scientific, USA). After 20 min incubation in a tissue culture incubator, the resulting cell suspension was removed from the limbal pieces and epithelial medium was added to this suspension. The cell suspension was centrifuged for 3 min at 1000 rpm in Heraeus Megafuge 16R Centrifuge (Thermo Fisher Scientific, USA), the supernatant removed and the remaining cell pellet re-suspended in epithelial medium containing 3:1 mixture of low-glucose DMEM:F12 supplemented with fetal calf serum 10%, penicillin/streptomycin 1% (all Thermo Fisher Scientific, USA), hydrocortisone 0.4 μg/ml, insulin 5 μg/ml, triiodothyronine 1.4 ng/ml, adenine 24 μg/ml, cholera toxin 8.4 ng/ml and EGF 10 ng/ml (all Sigma-Aldrich, UK). The trypsinization and centrifugation process was repeated a further three times using the same limbal tissue and the same centrifuge settings. The resulting cell suspensions were pooled together. Cells were counted and assessed for viability using trypan blue exclusion and a haemocytometer. 30,000 viable LECs in epithelial medium were added to one 9.6 cm^2^ tissue culture well containing the growth arrested 3T3 fibroblasts and placed in a tissue culture incubator at 37 °C with a humidified atmosphere containing 5% CO_2_. The medium was exchanged on the third culture day and every other day thereafter. Several days after, cell colonies with typical morphology started to appear and were cultured until they became sub-confluent. Following this 3T3 feeder cells were detached and removed using 0.02% EDTA (Lonza, Switzerland), sub-confluent primary cultures were dissociated with 0.5% trypsin-EDTA (Santa Cruz, USA) to single cell suspension and passaged at a density of 6 × 10^3^ cells/cm.^2^ For serial propagation, cells were passaged and cultured as above, always at the stage of sub-confluence, until they reached passage 3.

### Air liquid interface differentiations

Air liquid interface differentiations were performed by harvesting cultured human limbal epithelial cells as described above. 200,000 LECs were plated on Matrigel coated 24-well plate cell culture inserts (ThinCerts™, Greiner bio-one). Once confluent, the apical medium was removed, and the cells were fed from the basal chamber with epithelial medium up to one month.

### Human LECs culture with proinflammatory cytokines

LECs were plated on mitotically inactivated 3T3 at density of 3 × 10^3^ cells/cm2. The day after, LECs media was supplemented with IL6, TNFα or IL1β, all at concentration of 10 ng/ml. Daily media changes were performed and LECs were harvested at day 7.

### Human LECs siRNA transfection

Human LECs from 3 different donors were grown on 3T3 feeder layer in complete epithelial medium supplemented with EGF, adenine, cholera toxin, hydrocortisone, insulin, and triiodothyronine. A day before transfection, LECs (150 × 10^3^) were re-seeded in 12-well plate without feeders to increase transfection efficiency in EpiGrow Human Ocular Basal Medium. The day after re-seeding cells were transfected with either *GPHA2 (*HSS153169), or *TFPI2* (HSS111884) Human Stealth siRNAs and Stealth RNAi siRNA Negative Control Lo GC using Lipofectamine™ RNAiMAX Transfection Reagent (ThermoFisher Scientific) according to the manufacturer's protocol. After 48 h incubation with siRNA, cells we re-seeded into 6 well plates for colony forming efficiency and clonal assays and cultured on 3T3 feeders for 14 days. The rest of the cells were used for qRT-PCR.

### Colony forming efficiency (CFE) assay for human LECs

Mitotically inactivated 3T3-J2 mouse embryonic fibroblasts (Kerafast, USA) were suspended in complete medium containing: high-glucose DMEM (89%), FBS (10%) and penicillin/streptomycin (1%) and plated in a 9.6 cm^2^ tissue culture well at a final density of 2.4 × 10^4^ cells per cm^2^ and placed in a tissue culture incubator overnight to allow the establishment of a 3T3 feeder layer. The following day, 500 or 1000 viable LECs were plated onto the prepared 3T3 feeder cells together with 2 ml of epithelial medium. The CFE culture was then placed in the tissue culture incubator and the epithelial medium was changed on the third day and then every second day thereafter with regular microscopic examination (Eclipse TS100, Nikon, Japan) for the presence of colonies. The CFE was measured on the 12th day of the culture. This was performed by removal of the epithelial medium followed by two brief washes with PBS. The culture was then fixed with 3.7% formaldehyde (VWR International, UK) in PBS for 10 min at room temperature. Next, the formaldehyde solution was removed, and the culture was irrigated with PBS. The colonies were then stained by incubation with 1% Rhodamine B (Sigma-Aldrich) in methanol for 10 min at room temperature. Following staining, the colonies were counted under dissecting microscope (SMZ645, Nikon, Japan). The CFE was calculated using the formula: number of colonies formed/number of cells plated × 100.

### Clonal assays for human LECs

Clonal analysis was performed by plating 1000 cells on 10-cm dishes for 10–12 days. When colonies became visible, 15–20 randomly selected colonies were individually trypsinised and transferred to fresh mitotically inactivated 3T3 fibroblasts in complete epithelial media. The plates were fixed and stained with Rhodamine B after 12–14 days. The clonal type was determined by (1) the morphology and size of colonies and (2) the percentage of aborted colonies. Colonies that gave rise to <5% of aborted colonies were scored a holoclones [89].

### If of human LECs

Cultured LECs were fixed for 15 min in 4% PFA. A blocking step was performed by incubation in antibody diluent containing 1% bovine serum albumin (Sigma-Aldrich) with 5% normal goat serum (Thermo Fisher Scientific) for 30 min prior to staining. Permeabilization with 0.2% Triton X-100 in PBS was performed prior to staining with antibodies for internal cell markers. Cells were incubated with the appropriate primary antibodies (Table S14) at 4 °C overnight and further incubated with secondary antibodies for 1 h at room temperature. Following this, cells were washed and then mounted in Vectashield anti-fading media containing Hoechst (Vector Laboratories, UK). All details of antibody providers and concentrations are provided in Table S14.

### Image acquisition and processing

Adult and developmental eye sections and cultured LECs were viewed on a Zeiss Axio ImagerZ2 equipped with Apotome 2 and Zen 2012 blue software (Zeiss, Germany). Objectives lens used were EC Plan Neofluar 20x/0.5 Ph2 and EC Plan Apochromat 63x/1.4 Ph3. Series of XZ optical sections (<1 μm thick) were taken at 1.0 μm steps throughout the depth of the section. Final images are presented as a maximum projection and adjusted for brightness and contrast using the Zen software.

### Flow cytometric analysis and cell sorting

LECs were harvested as described above. 1 × 10^6^ single cells were resuspended in 100 μl FACS buffer (PBS supplemented with 5% FBS) and stained by incubation with 5 μl GPHA2 antibody (Santa Cruz, sc-390194 AF488) for 30 min at 4 °C in the dark. The cells were washed twice with PBS before resuspension in 1 ml FACS buffer for FACS experiment on a BD Fortessa Flow Cytometer. At least 20,000 cells were analysed in each run and data were analysed with FACSDiva software (BD Biosciences). Flow activated cell sorting was performed on a BD FACSAria.

### Quantitative reverse transcriptase polymerase chain reaction (qRT- PCR)

RNA was extracted from cultured LECs using the ReliaPrep RNA Cell Miniprep System (Promega). cDNA was then synthesised using the GoScript Reverse Transcription System as per the manufacturer's protocol. qPCR was then performed using Go-Taq qPCR Master Mix (Promega) and was composed of 5 μl GoTaq, 0.5 μl forward primer, 0.5 μl reverse primer, 0.5 μl template cDNA, 3.4 μl RNAse-free water and 0.1 μl CXR. All reactions were analysed on a QuantStudio™ 7 Flex Real Time PCR System (ThermoFisher Scientific) according to the manufacturer's instructions. A standard, 40-cycle qPCR was performed for each sample. The primer sequences used for qRT-PCR are listed in Table S15. The data was analysed using the 2-^ΔΔCt^ method.

## Data availability

All single cell data are deposited in the Gene Expression Omnibus under the accession number GSE155683. All scRNA-Seq datasets can be assessed and visualised through the interactive cell browser can be found in http://retinalstemcellresearch.co.uk/CorneaCellAtlas.

## Author contributions

JC: setup and optimisation of scRNA- and ATAC-Seq and tissue dissociations, performed experiments, experimental design, data collection and data submission.

RQ: setup bioinformatics pipeline, performed bioinformatics analysis, figure preparation and data submission.

DZ: performed IF experiments, data collection, IF data analysis, IF figure preparation.

BD: performed IF experiments, data collection, IF data analysis, IF figure preparation.

SB: performed RNAi experiments, data analysis and figure preparation.

NM, MMM, SD, CY, GR and JMC: performed experiments, data collection.

GR, MH: data analysis.

SL, DH: facilitated embryonic and fetal sample collection for the study.

AJ, PR, SG, LC: facilitated adult sample collection for the study.

CC, FF: experimental design and fund raising.

LA: performed experiments, data collection, data analysis, figure preparation, experimental design, and manuscript writing.

ML: designed and performed experiments, data collection and analysis, figure preparation, manuscript writing and fund raising.

All authors contributed and approved the final version of the manuscript.

## Declaration of competing interest

None.

## Figures and Tables

**figs1:**
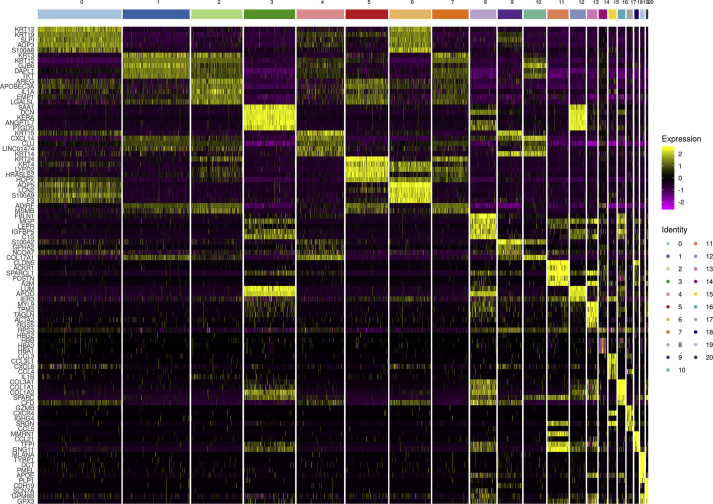


**figs2:**
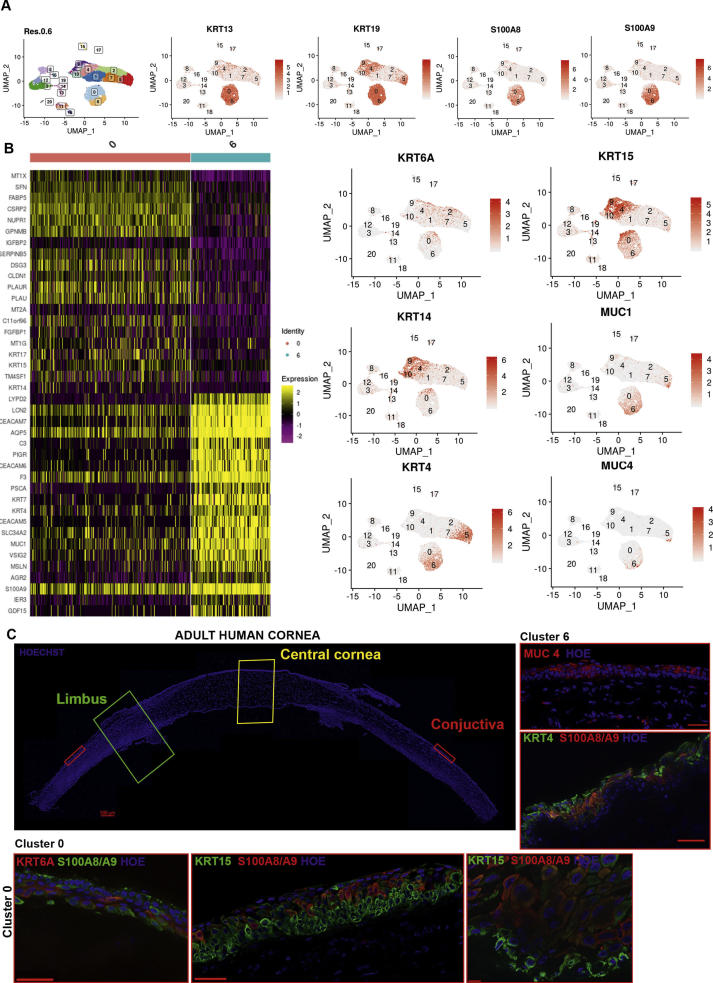


**figs3:**
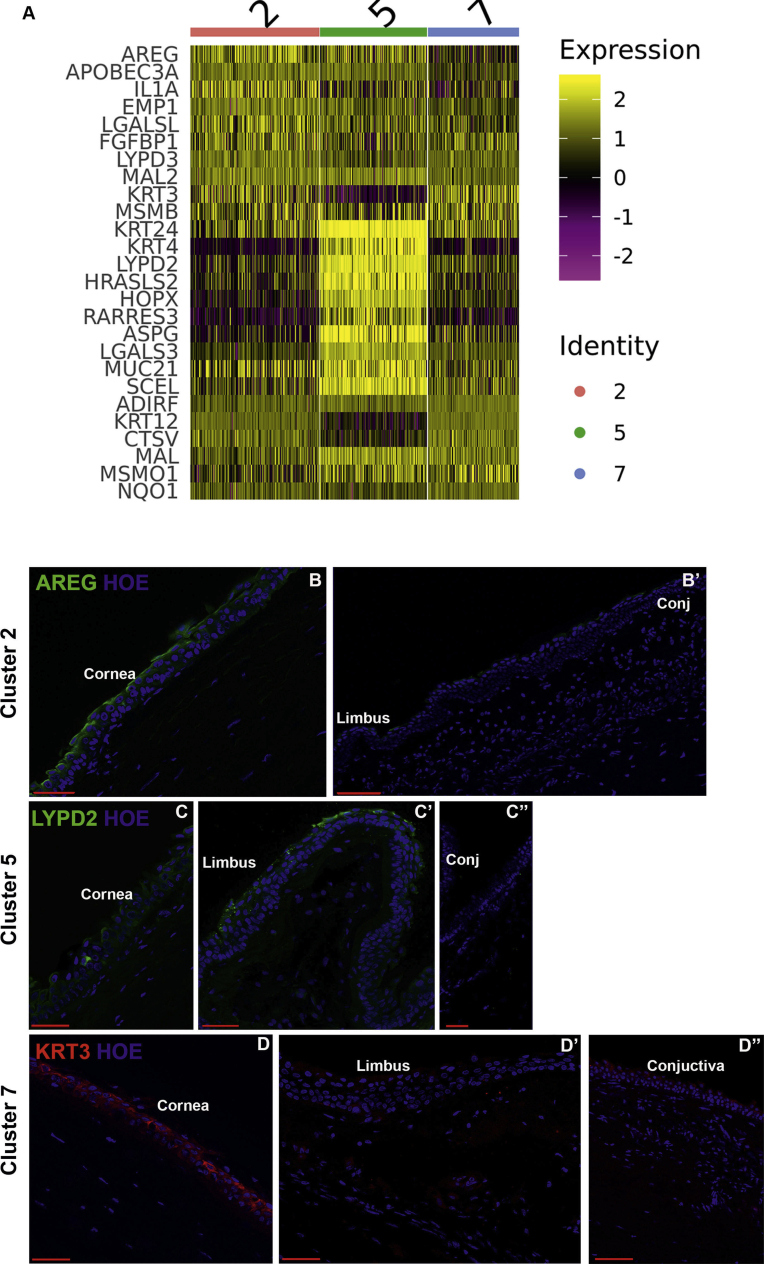


**figs4:**
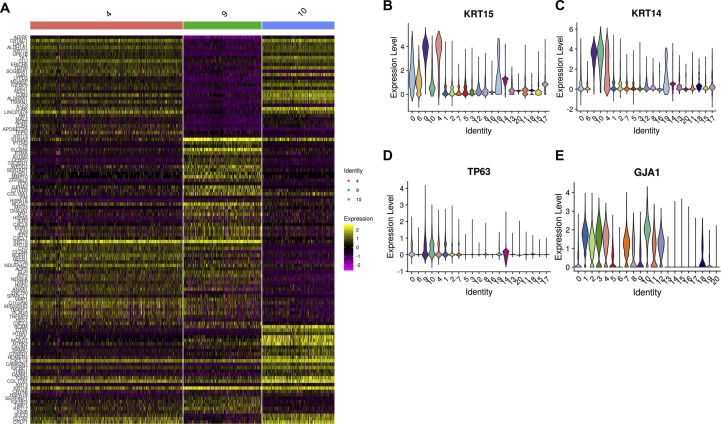


**figs5:**
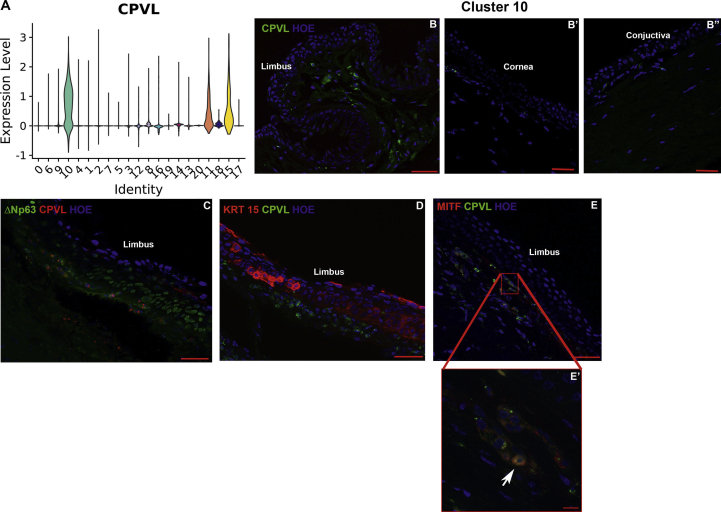


**figs6:**
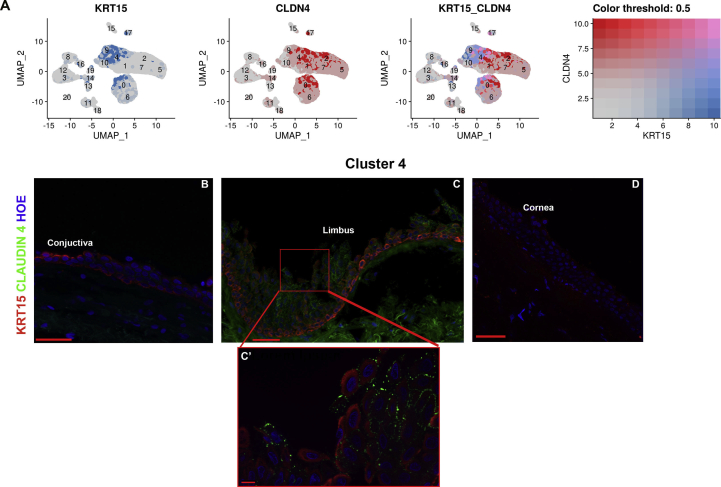


**figs7:**
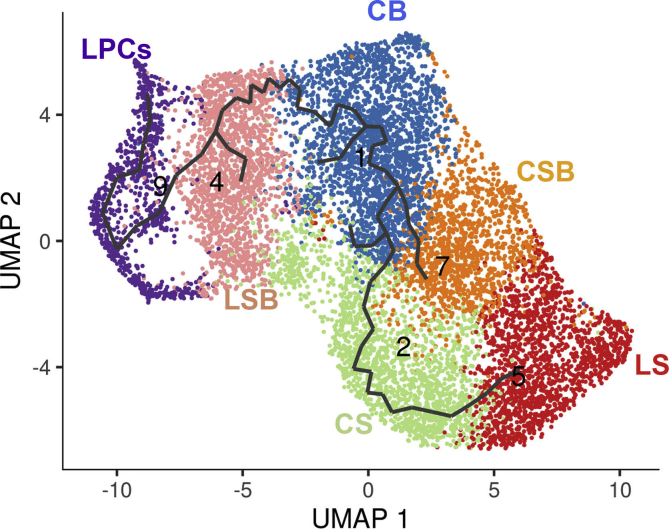


**figs8:**
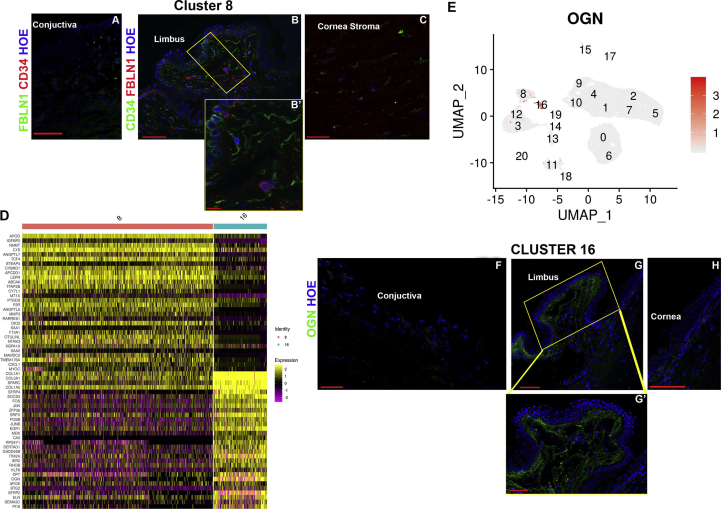


**figs9:**
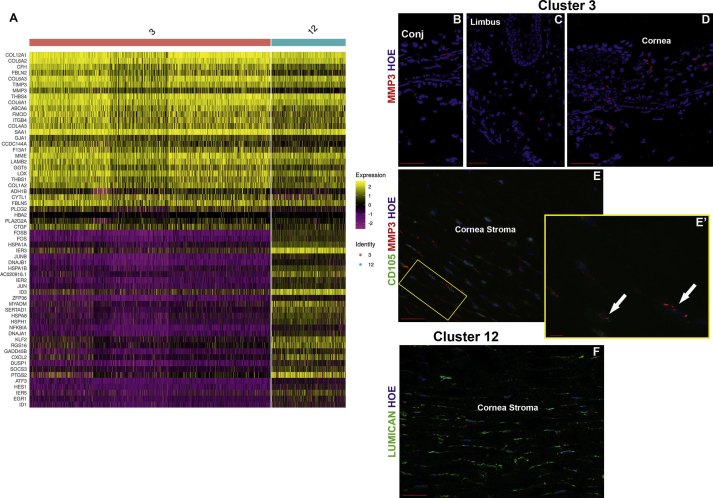


**figs10:**
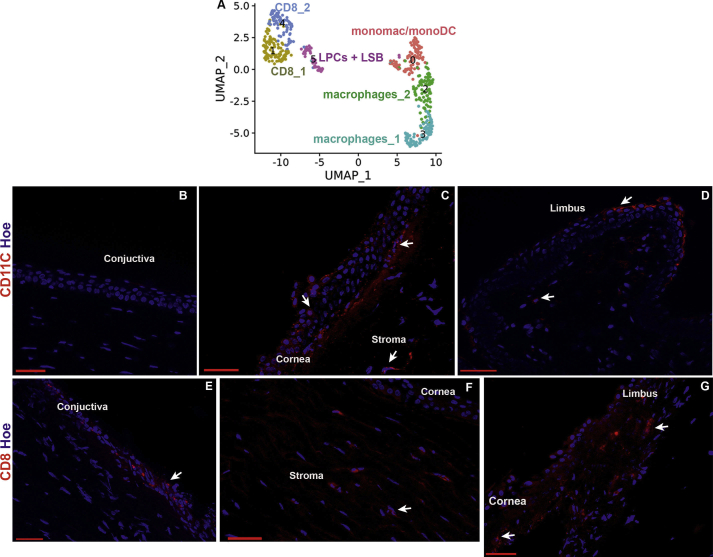


**figs11:**
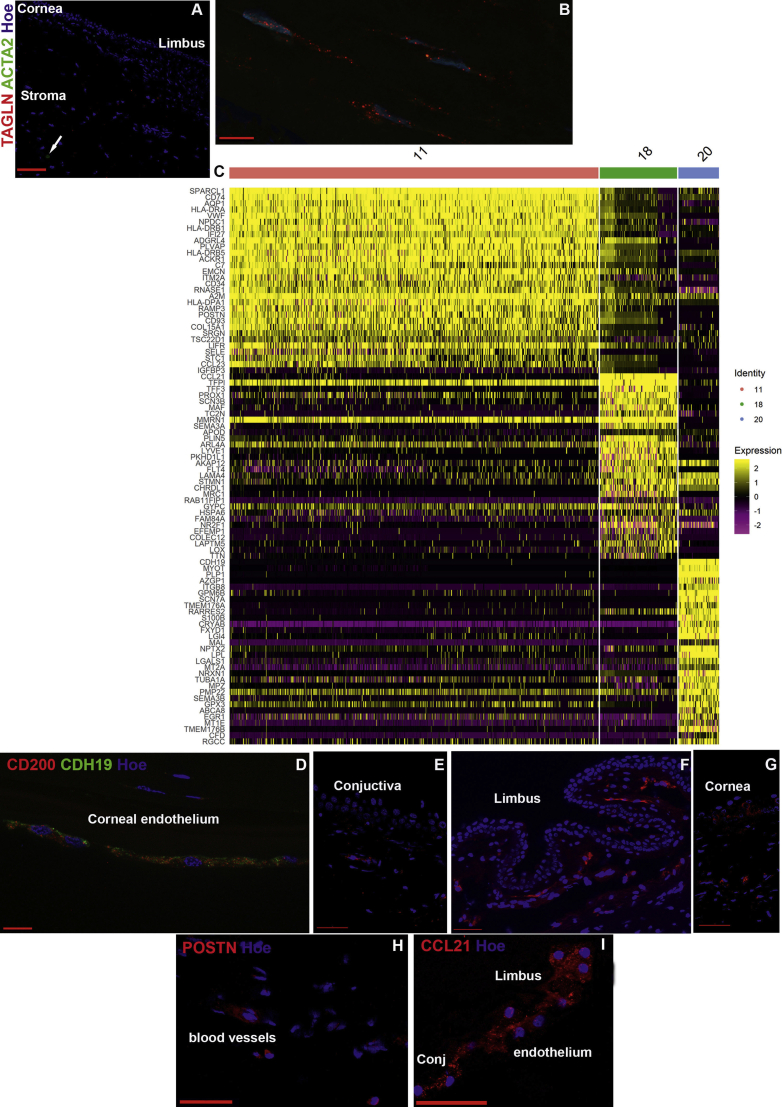


**figs12:**
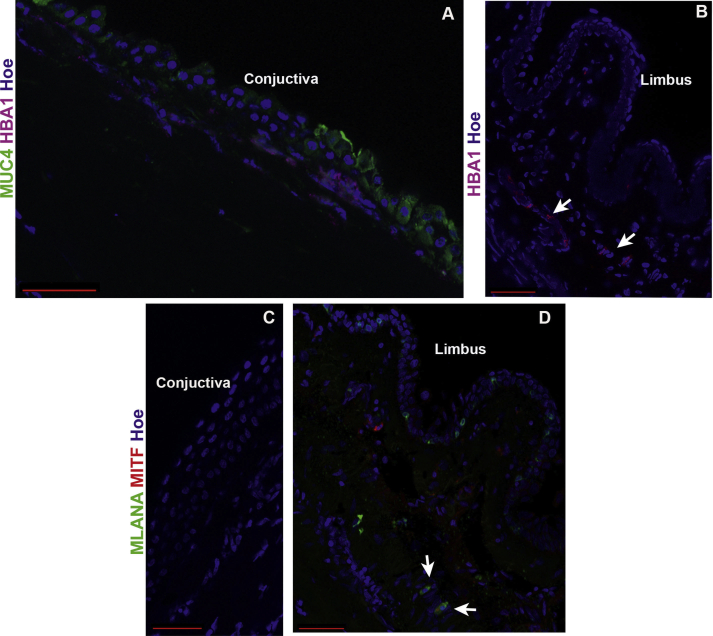


**figs13:**
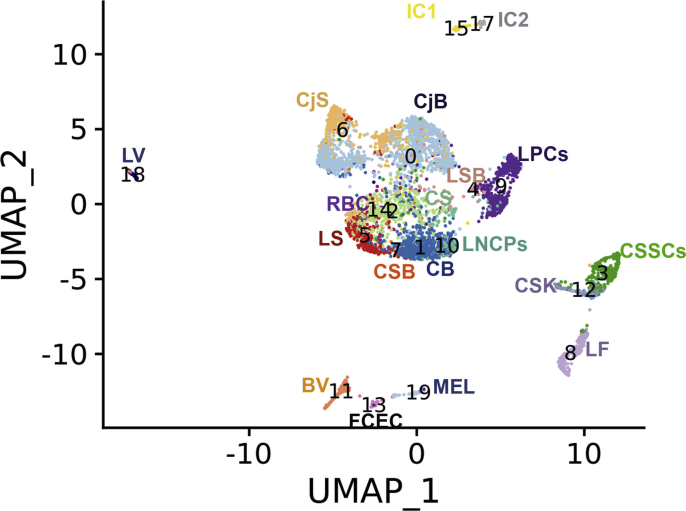


**figs14:**
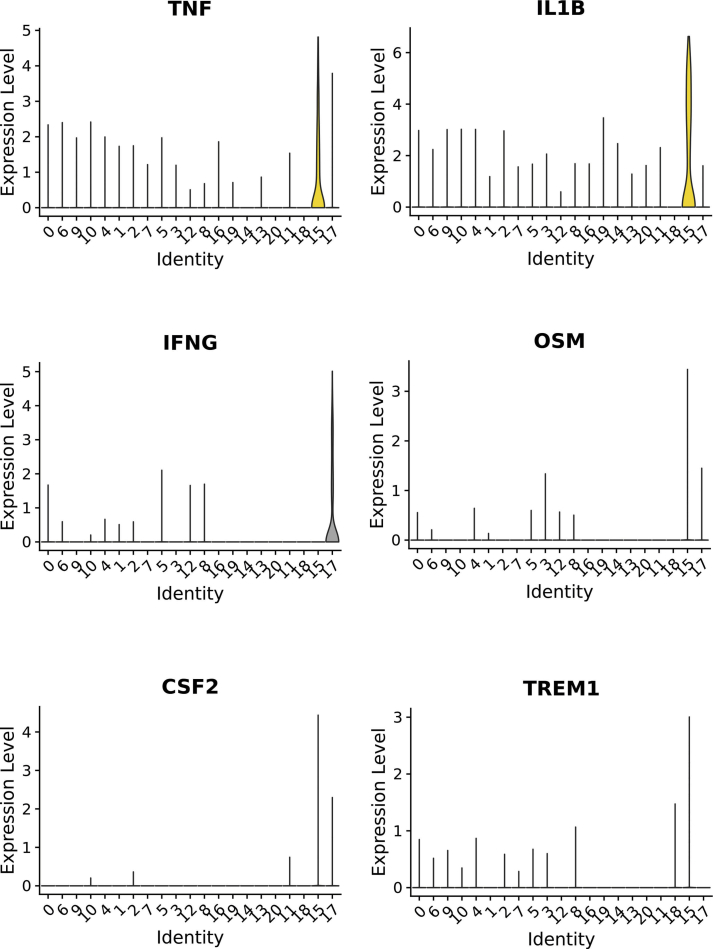


**figs15:**
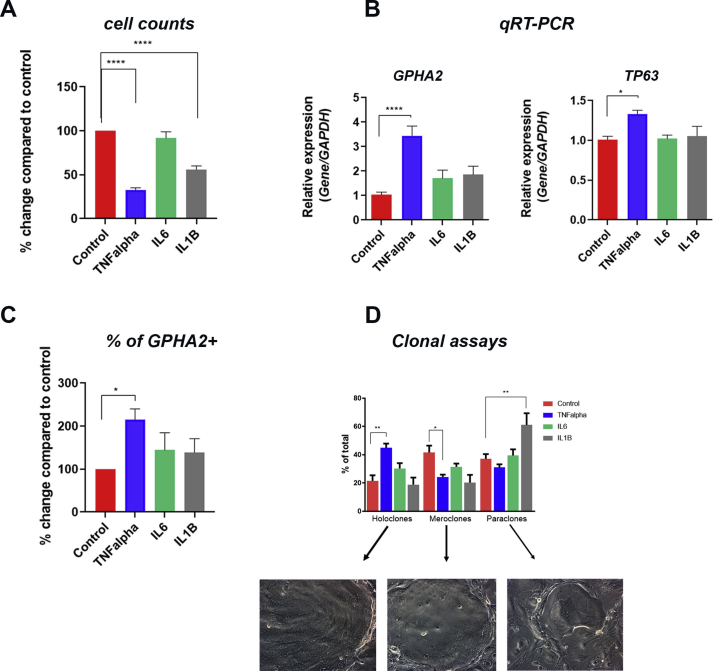


**figs16:**
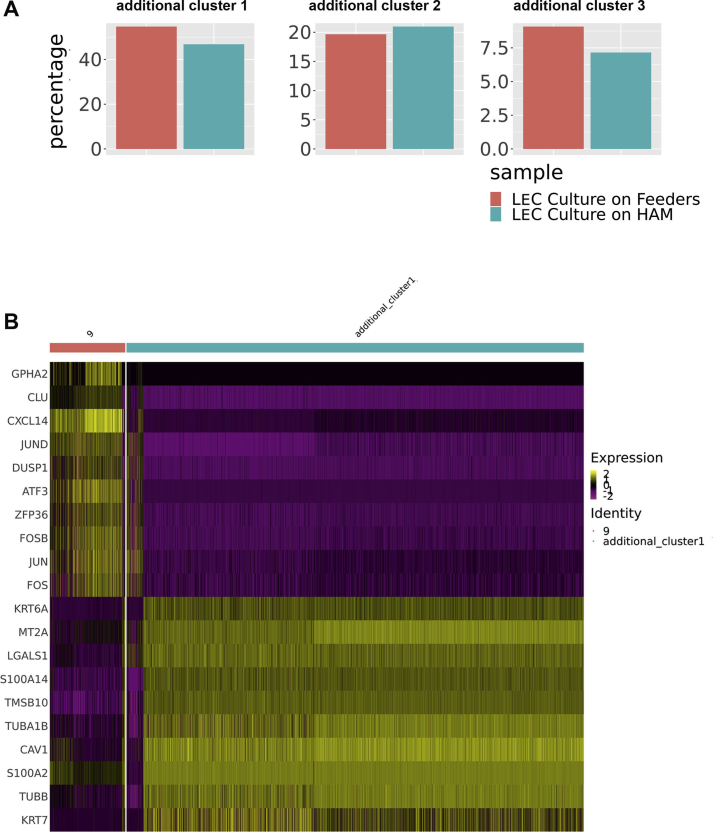


**figs17:**
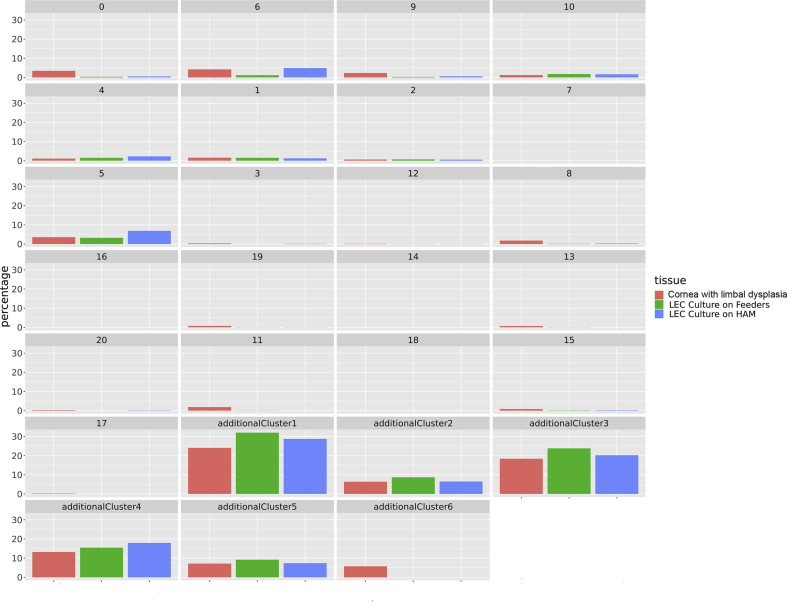


**figs18:**
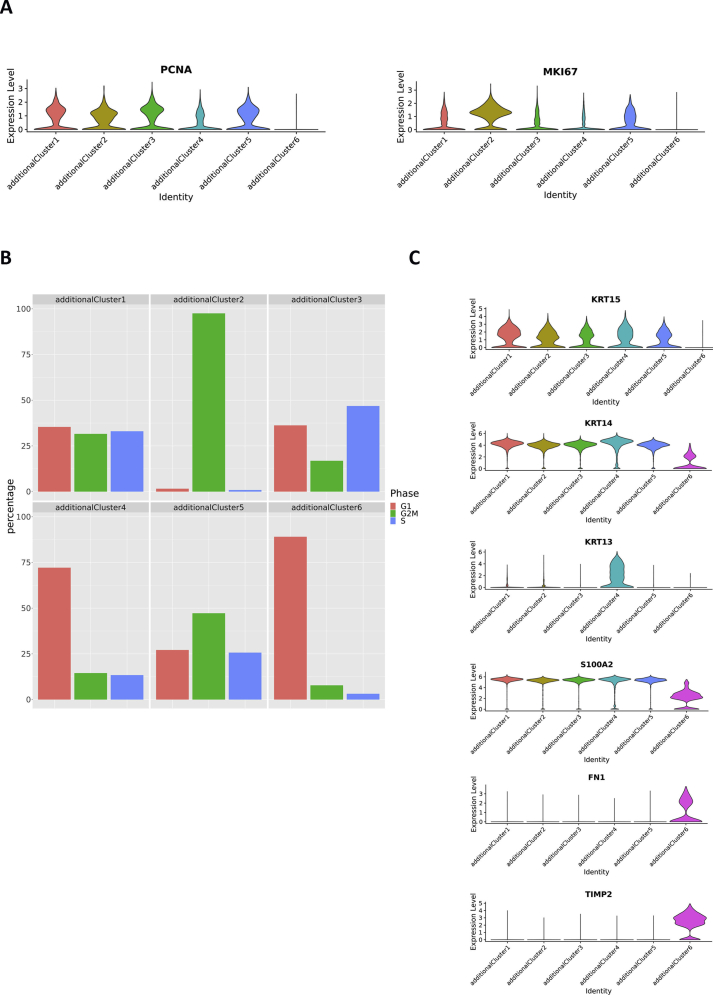


**figs19:**
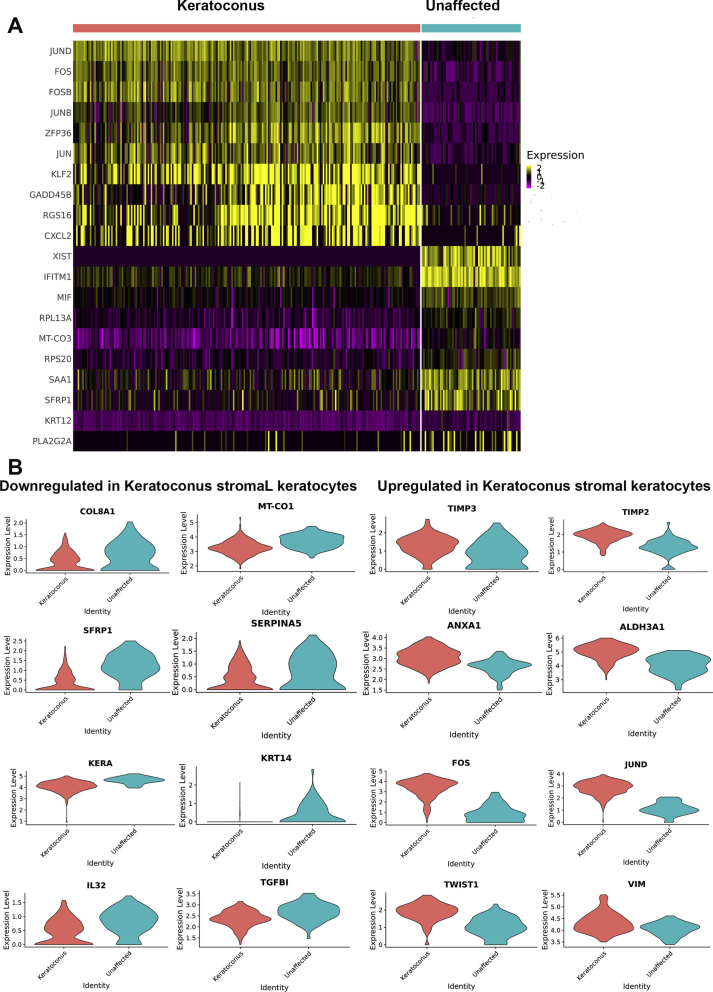


**figs20:**
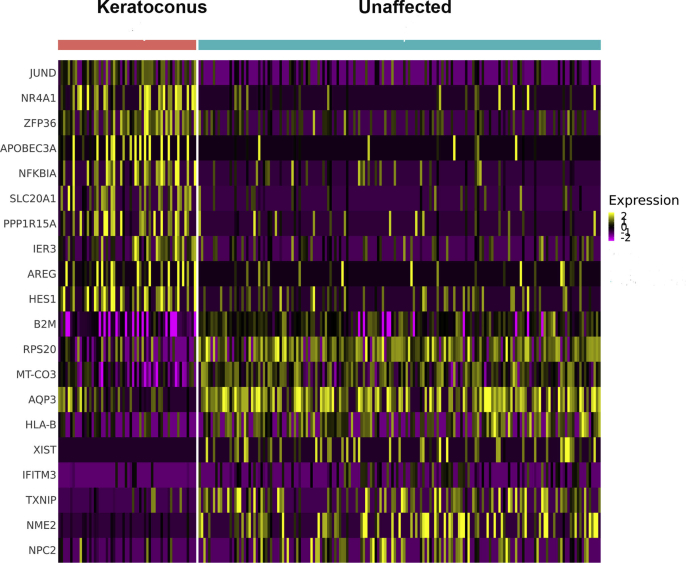


**figs21:**
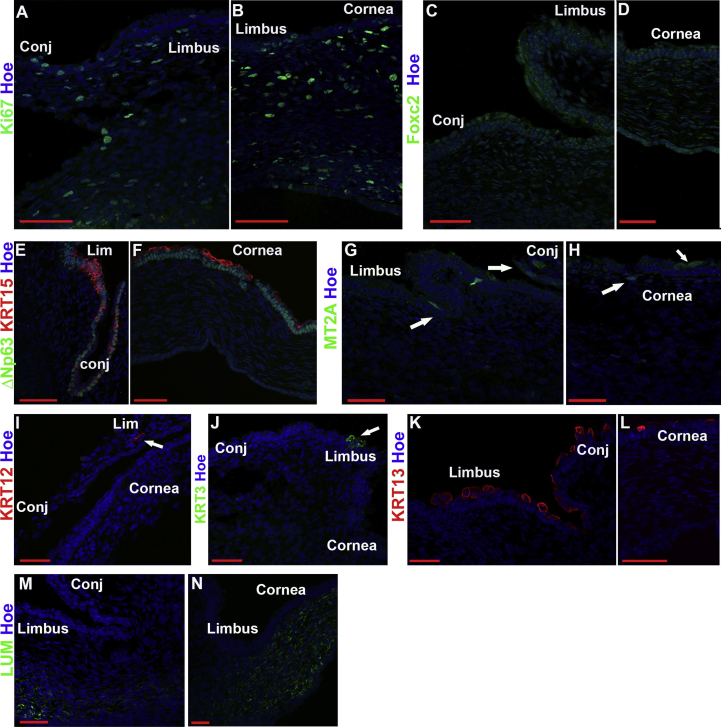


**figs22:**
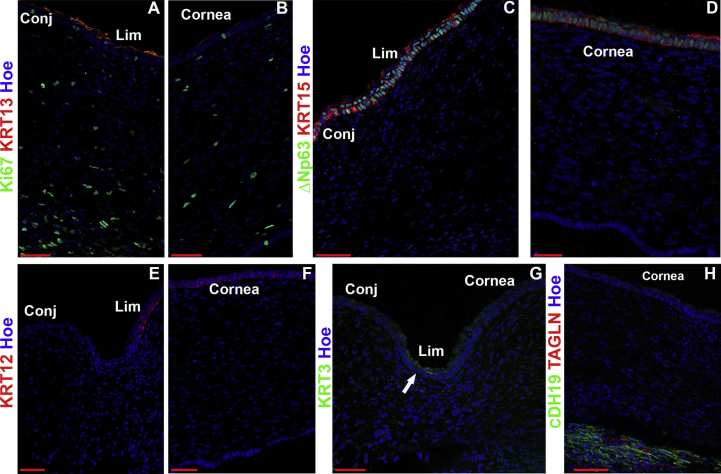


**figs23:**
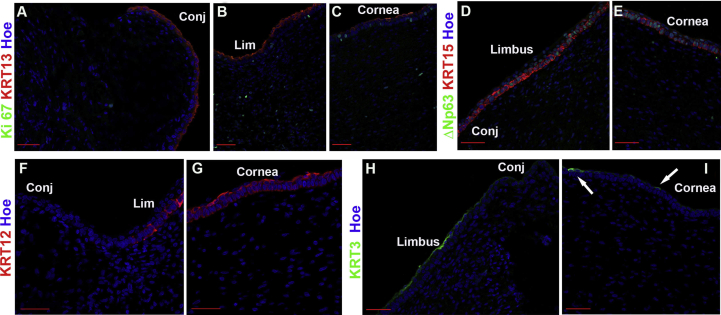


**figs24:**
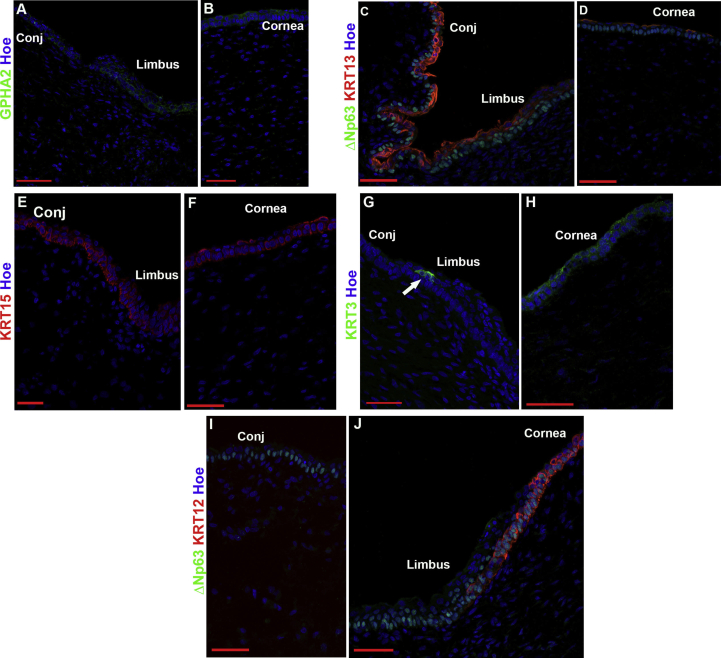


**figs25:**
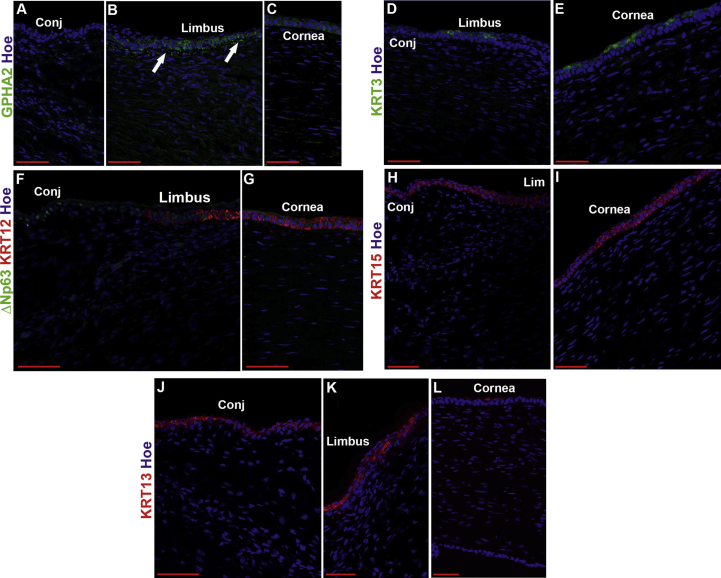


**figs26:**
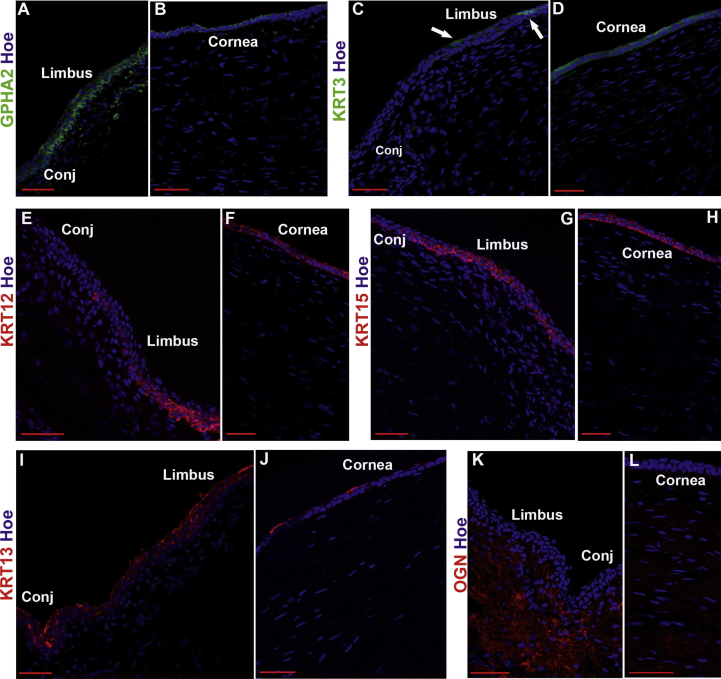


## Abbreviations

CEND –: corneal endothelium
EBM: epithelial basement membrane
Ep –: epithelial
LV –: lymphatic vessels
FCEC –: fibroblastic corneal endothelial cells
PEP –: proliferating epithelial progenitors
Prolif –: proliferating
Prog –: progenitors

## References

[1] Osei-Bempong C., Figueiredo F.C., Lako M. (2013). The limbal epithelium of the eye - a review of limbal stem cell biology, disease and treatment. Bioessays.

[2] Oliva M.S., Schottman T., Gulati M. (2012). Turning the tide of corneal blindness. Indian J Ophthalmol.

[3] Pascolini D., Mariotti S.P. (2012). Global estimates of visual impairment: 2010. Br J Ophthalmol.

[4] Whitcher J.P., Srinivasan M., Upadhyay M.P. (2001). Corneal blindness: a global perspective. Bull World Health Organ.

[5] Biomaterials and Regenerative Medicine in Ophthalmology - 1st Edition n.d. https://www.elsevier.com/books/biomaterials-and-regenerative-medicine-in-ophthalmology/chirila/978-1-84569-443-2 (accessed June 29, 2020).

[6] Kolli S., Ahmad S., Mudhar H.S., Meeny A., Lako M., Figueiredo F.C. (2014). Successful application of ex vivo expanded human autologous oral mucosal epithelium for the treatment of total bilateral limbal stem cell deficiency. Stem Cell.

[7] Kolli S., Ahmad S., Lako M., Figueiredo F. (2009). Successful clinical implementation of corneal epithelial stem cell therapy for treatment of unilateral limbal stem cell deficiency. Stem Cell.

[8] Sridhar M.S. (2018). Anatomy of cornea and ocular surface. Indian J Ophthalmol.

[9] Cotsarelis G., Cheng S.-Z., Dong G., Sun T.-T., Lavker’ R.M. (1989). Existence of slow-cycling limbal epithelial basal cells that can Be preferentially stimulated to proliferate: implications on epithelial. Stem Cell.

[10] Hertsenberg A.J., Funderburgh J.L. (2015). Stem cells in the cornea. Prog. Mol. Biol. Transl. Sci..

[11] Pinnamaneni N., Funderburgh J.L. (2012). Concise review: stem cells in the corneal stroma. Stem Cell.

[12] Lwigale P.Y. (2015). Corneal development: different cells from a common progenitor. Prog. Mol. Biol. Transl. Sci..

[13] Zheng T., Le Q., Hong J., Xu J. (2016). Comparison of human corneal cell density by age and corneal location: an in vivo confocal microscopy study. BMC Ophthalmol.

[14] Kaplan N., Wang J., Wray B., Patel P., Yang W., Peng H. (2019). Single-cell RNA transcriptome helps define the limbal/corneal epithelial stem/early transit amplifying cells and how autophagy affects this population. Invest Ophthalmol Vis Sci.

[15] Li D.Q., Kim S., Li J.M., Gao Q., Choi J., Bian F. (2021). Single-cell transcriptomics identifies limbal stem cell population and cell types mapping its differentiation trajectory in limbal basal epithelium of human cornea. Ocul Surf.

[16] Bargagna‐Mohan P., Schultz G., Rheaume B., Trakhtenberg E.F., Robson P., Pal‐Ghosh S. (2021). Corneal nonmyelinating Schwann cells illuminated by single‐cell transcriptomics and visualized by protein biomarkers. J Neurosci Res.

[17] Merjava S., Neuwirth A., Tanzerova M., Jirsova K. (2011). The spectrum of cytokeratins expressed in the adult human cornea, limbus and perilimbal conjunctiva. Histol Histopathol.

[18] Cytokeratin 13 is a Marker for Human Conjunctival Epithelium | IOVS | ARVO Journals n.d. https://iovs.arvojournals.org/article.aspx?articleid=2352846 (accessed June 29, 2020).

[19] Li J., Riau A.K., Setiawan M., Mehta J.S., Ti S.E., Tong L. (2011). S100A expression in normal corneal-limbal epithelial cells and ocular surface squamous cell carcinoma tissue. Mol Vis.

[20] Tanifuji-Terai N., Terai K., Hayashi Y., Chikama T.I., Kao W.W.Y. (2006). Expression of keratin 12 and maturation of corneal epithelium during development and postnatal growth. Investig Ophthalmol Vis Sci.

[21] Shurman D.L., Glazewski L., Gumpert A., Zieske J.D., Richard G. (2005). In vivo and in vitro expression of connexins in the human corneal epithelium. Investig Ophthalmol Vis Sci.

[22] Djalilian A.R., Namavari A., Ito A., Balali S., Afshar A., Lavker R.M. (2008). Down-regulation of Notch signaling during corneal epithelial proliferation.

[23] Chen B., Mi S., Wright B., Connon C.J. (2010). Investigation of K14/K5 as a stem cell marker in the limbal region of the bovine cornea. PloS One.

[24] Yoshida S., Shimmura S., Kawakita T., Miyashita H., Den S., Shimazaki J. (2006). Cytokeratin 15 can be used to identify the limbal phenotype in normal and diseased ocular surfaces. Investig Ophthalmol Vis Sci.

[25] Chen S.Y., Cheng A.M.S., Zhang Y., Zhu Y.T., He H., Mahabole M. (2019). Pax 6 controls neural crest potential of limbal niche cells to support self-renewal of limbal epithelial stem cells. Sci Rep.

[26] Schlotzer-Schrehardt U., Kruse F. (2005). Identification and characterisation of limbal stem cells. Exp Eye Res.

[27] Yoshida Y., Ban Y., Kinoshita S. (2009). Tight junction transmembrane protein claudin subtype expression and distribution in human corneal and conjunctival epithelium. Investig Ophthalmol Vis Sci.

[28] Wang W., Li S., Xu L., Jiang M., Li X., Zhang Y. (2020). Differential gene expression between limbal niche progenitors and bone marrow derived mesenchymal stem cells. Int J Med Sci.

[29] Nakayasu K., Tanaka M., Konomi H., Hayashi T. (1986). Distribution of types I, II, III, IV and V collagen in normal and keratoconus corneas. Ophthalmic Res.

[30] Kao W.W.Y., Liu C.Y. (2002). Roles of lumican and keratocan on corneal transparency. Glycoconj J.

[31] Hashmani K., Branch M.J., Sidney L.E., Dhillon P.S., Verma M., McIntosh O.D. (2013). Characterization of corneal stromal stem cells with the potential for epithelial transdifferentiation. Stem Cell Res Ther.

[32] Palomar AP del, Montolío A., Cegoñino J., Dhanda S.K., Lio C.T., Bose T. (2019). The innate immune cell profile of the cornea predicts the onset of ocular surface inflammatory disorders. J Clin Med.

[33] Li Z., Burns A.R., Rumbaut R.E., Smith C.W. (2007). γδ T cells are necessary for platelet and neutrophil accumulation in limbal vessels and efficient epithelial repair after corneal abrasion. Am J Pathol.

[34] Roy O., Leclerc V.B., Bourget J.M., Thériault M., Proulx S. (2015). Understanding the process of corneal endothelial morphological change in vitro. Investig Ophthalmol Vis Sci.

[35] Kay E.P., Smith R.E., Nimniage M.E. (1985).

[36] Thiriot A., Perdomo C., Cheng G., Novitzky-Basso I., McArdle S., Kishimoto J.K. (2017). Differential DARC/ACKR1 expression distinguishes venular from non-venular endothelial cells in murine tissues. BMC Biol.

[37] Liu L., Nielsen F.M., Emmersen J., Bath C., Østergaard Hjortdal J., Riis S. (2018). Pigmentation is associated with stemness hierarchy of progenitor cells within cultured limbal epithelial cells. Stem Cell.

[38] Cao Q., Anyansi C., Hu X., Xu L., Xiong L., Tang W. (2017). Reconstruction of enhancer-target networks in 935 samples of human primary cells, tissues and cell lines. Nat Genet.

[39] Efremova M., Vento-Tormo M., Teichmann S.A., Vento-Tormo R. (2020). CellPhoneDB: inferring cell–cell communication from combined expression of multi-subunit ligand–receptor complexes. Nat Protoc.

[40] Yang L., Zhang S., Duan H., Dong M., Hu X., Zhang Z. (2019). Different effects of pro-inflammatory factors and hyperosmotic stress on corneal epithelial stem/progenitor cells and wound healing in mice. Stem Cells Transl Med.

[41] Puri S., Sun M., Mutoji K.N., Gesteira T.F., Coulson-Thomas V.J. (2020). Epithelial cell migration and proliferation patterns during initial wound closure in normal mice and an experimental model of limbal stem cell deficiency. Investig Ophthalmol Vis Sci.

[42] Veréb Z., Albert R., Póliska S., Olstad O.K., Akhtar S., Moe M.C. (2013). Comparison of upstream regulators in human ex vivo cultured cornea limbal epithelial stem cells and differentiated corneal epithelial cells. BMC Genom.

[43] Notara M., Shortt A.J., Galatowicz G., Calder V., Daniels J.T. (2010). IL6 and the human limbal stem cell niche: a mediator of epithelial-stromal interaction. Stem Cell Res.

[44] Trosan P., Svobodova E., Chudickova M., Krulova M., Zajicova A., Holan V. (2012). The key role of insulin-like growth factor i in limbal stem cell differentiation and the corneal wound-healing process. Stem Cell Dev.

[45] Meyer-Blazejewska E.A., Kruse F.E., Bitterer K., Meyer C., Hofmann-Rummelt C., Wünsch P.H. (2010). Preservation of the limbal stem cell phenotype by appropriate culture techniques. Investig Ophthalmol Vis Sci.

[46] Wilson S.E., He Y.G., Weng J., Zieske J.D., Jester J.V., Schultz G.S. (1994). Effect of epidermal growth factor, hepatocyte growth factor, and keratinocyte growth factor, on proliferation, motility and differentiation of human corneal epithelial cells. Exp Eye Res.

[47] Wasson Jdz M. Regional variation in distribution of EGF receptor in developing and adult corneal epithelium - PubMed 1993:145–52. https://pubmed.ncbi.nlm.nih.gov/8270620/.

[48] Nakamura Y., Sotozono C., Kinoshita S. (2001). The epidermal growth factor receptor (EGFR): role in corneal wound healing and homeostasis. Exp Eye Res.

[49] Zhang J., Upadhya D., Lu L., Reneker L.W. (2015). Fibroblast growth factor receptor 2 (FGFR2) is required for corneal epithelial cell proliferation and differentiation during embryonic development. PloS One.

[50] Ueno C., Ferrari G., Hattori T., Saban D.R., Katikireddy K.R., Chauhan S.K. (2012). Dependence of corneal stem/progenitor cells on ocular surface innervation “dependence of corneal stem/progenitor cells on ocular surface innervation. Investigative Opthalmology & Visual Science.

[51] Kolli S., Bojic S., Ghareeb A.E., Kurzawa-Akanbi M., Figueiredo F.C., Lako M. (2019). The role of nerve growth factor in maintaining proliferative capacity, colony-forming efficiency, and the limbal stem cell phenotype. Stem Cell.

[52] Sacchetti M., Lambiase A. (2014). Diagnosis and management of neurotrophic keratitis. Clin Ophthalmol.

[53] Cursiefen C., Rummelt C., Küchle M. (2000). Immunohistochemical localization of vascular endothelial growth factor, transforming growth factor α, and transforming growth factor β1 in human corneas with neovascularization. Cornea.

[54] Cursiefen C., Masli S., Ng T.F., Dana M.R., Bornstein P., Lawler J. (2004). Roles of thrombospondin-1 and -2 in regulating corneal and iris angiogenesis. Investig Ophthalmol Vis Sci.

[55] Baylis O., Figueiredo F., Henein C., Lako M., Ahmad S. (2011). 13 Years of cultured limbal epithelial cell therapy: a review of the outcomes. J Cell Biochem.

[56] Stuart T., Butler A., Hoffman P., Hafemeister C., Papalexi E., Mauck W.M. (2019). Comprehensive integration of single-cell data. Cell.

[57] Thomas P.B., Liu Y.H., Zhuang F.F., Selvam S., Song S.W., Smith R.E. (2007). Identification of Notch-1 expression in the limbal basal epithelium. Mol Vis.

[58] Schlötzer-Schrehardt U., Kruse F.E. (2005). Identification and characterization of limbal stem cells. Exp Eye Res.

[59] Ramirez-Miranda A., Nakatsu M.N., Zarei-Ghanavati S., Nguyen C.V., Deng S.X. (2011). Keratin 13 is a more specific marker of conjunctival epithelium than keratin 19. Mol Vis.

[60] Djurec M., Graña O., Lee A., Troulé K., Espinet E., Cabras L. (2018). Saa3 is a key mediator of the protumorigenic properties of cancer-associated fibroblasts in pancreatic tumors. Proc Natl Acad Sci U S A.

[61] Liu L., Walker E.A., Kissane S., Khan I., Murray P.I., Rauz S. (2011). Gene expression and miR profiles of human corneal fibroblasts in response to dexamethasone. Investig Ophthalmol Vis Sci.

[62] Mas Tur V., MacGregor C., Jayaswal R., O'Brart D., Maycock N. (2017). A review of keratoconus: diagnosis, pathophysiology, and genetics. Surv Ophthalmol.

[63] Khaled M.L., Helwa I., Drewry M., Seremwe M., Estes A., Liu Y. (2017). Molecular and histopathological changes associated with keratoconus.

[64] Chaerkady R., Shao H., Scott S.G., Pandey A., Jun A.S., Chakravarti S. (2013). The keratoconus corneal proteome: loss of epithelial integrity and stromal degeneration. J Proteomics.

[65] Shinde V., Hu N., Renuse S., Mahale A., Pandey A., Eberhart C. (2019). Mapping keratoconus molecular substrates by multiplexed high-resolution proteomics of unpooled corneas. OMICS A J Integr Biol.

[66] Vallabh N.A., Romano V., Willoughby C.E. (2017). Mitochondrial dysfunction and oxidative stress in corneal disease. Mitochondrion.

[67] Wojcik K.A., Kaminska A., Blasiak J., Szaflik J., Szaflik J.P. (2013). Oxidative stress in the pathogenesis of keratoconus and Fuchs endothelial corneal dystrophy. Int J Mol Sci.

[68] Kao W.W.Y., Vergnes J.P., Ebert J., Sundar-Raj C.V., Brown S.I. (1982). Increased collagenase and gelatinase activities in keratoconus. Biochem Biophys Res Commun.

[69] McMonnies C.W. (2015). Inflammation and keratoconus. Optom Vis Sci.

[70] Davies S.B., Chui J., Madigan M.C., Provis J.M., Wakefield D., Di Girolamo N. (2009). Stem cell activity in the developing human cornea. Stem Cell.

[71] Rodrigues M., Ben-Zvi A., Krachmer J., Schermer A., Sun T.T. (1987). Suprabasal expression of a 64-kilodalton keratin (no. 3) in developing human corneal epithelium. Differentiation.

[72] Gage P.J., Rhoades W., Prucka S.K., Hjalt T. (2005). Fate maps of neural crest and mesoderm in the mammalian eye. Investig Ophthalmol Vis Sci.

[73] Sevel D., Isaacs R., Ferry A.P. (1988). A re-evaluation of corneal development. Trans Am Ophthalmol Soc.

[74] Behjati S., Lindsay S., Teichmann S.A., Haniffa M. (2018). Mapping human development at single-cell resolution. Dev.

[75] Braunger B.M., Ademoglu B., Koschade S.E., Fuchshofer R., Gabelt B.T., Kiland J.A. (2014). Identification of adult stem cells in Schwalbe's line region of the primate eye. Invest Ophthalmol Vis Sci.

[76] Baylis O., Rooney P., Figueiredo F., Lako M., Ahmad S. (2013). An investigation of donor and culture parameters which influence epithelial outgrowths from cultured human cadaveric limbal explants. J Cell Physiol.

[77] Holoclar European medicines agency. https://www.ema.europa.eu/en/medicines/human/EPAR/holoclar.

[78] Rama P., Matuska S., Paganoni G., Spinelli A., De Luca M., Pellegrini G. (2010). Limbal stem-cell therapy and long-term corneal regeneration. N Engl J Med.

[79] Mort R.L., Douvaras P., Morley S.D., Dorà N., Hill R.E., Collinson J.M. (2012). Stem cells and corneal epithelial maintenance: insights from the mouse and other animal models. Results Probl Cell Differ.

[80] Barbaro V., Testa A., Di Iorio E., Mavilio F., Pellegrini G., De Luca M. (2007). C/EBPδ regulates cell cycle and self-renewal of human limbal stem cells. J Cell Biol.

[81] Clayton E., Doupé D.P., Klein A.M., Winton D.J., Simons B.D., Jones P.H. (2007). A single type of progenitor cell maintains normal epidermis. Nature.

[82] Snippert H.J., van der Flier L.G., Sato T., van Es J.H., van den Born M., Kroon-Veenboer C. (2010). Intestinal crypt homeostasis results from neutral competition between symmetrically dividing Lgr5 stem cells. Cell.

[83] Giroux V., Lento A.A., Islam M., Pitarresi J.R., Kharbanda A., Hamilton K.E. (2017). Long-lived keratin 15+ esophageal progenitor cells contribute to homeostasis and regeneration. J Clin Invest.

[84] Altshuler A., Amitai-Lange A., Tarazi N., Dey S., Strinkovsky L., Bhattacharya S. (2020). Capturing limbal epithelial stem cell population dynamics, signature, and their niche. BioRxiv.

[85] Okada S.L., Ellsworth J.L., Durnam D.M., Haugen H.S., Holloway J.L., Kelley M.L. (2006). A glycoprotein hormone expressed in corticotrophs exhibits unique binding properties on thyroid-stimulating hormone receptor. Mol Endocrinol.

[86] Gerrelli D., Lisgo S., Copp A.J., Lindsay S. (2015). Enabling research with human embryonic and fetal tissue resources. Dev.

[87] Yu M., Bojic S., Figueiredo G.S., Rooney P., de Havilland J., Dickinson A. (2016). An important role for adenine, cholera toxin, hydrocortisone and triiodothyronine in the proliferation, self-renewal and differentiation of limbal stem cells in vitro. Exp Eye Res.

[88] Ahmad S., Stewart R., Yung S., Kolli S., Armstrong L., Stojkovic M. (2007). Differentiation of human embryonic stem cells into corneal epithelial-like cells by in vitro replication of the corneal epithelial stem cell niche. Stem Cell.

[89] Barrandon Y., Green H. (1987). Three clonal types of keratinocyte with different capacities for multiplication. Proc Natl Acad Sci U S A.

