# Supplementary material for: A single cell atlas of human cornea that defines its development, limbal progenitor cells and their interactions with the immune cells
Source: Ocul Surf. 2021 Jul;21:279–98. doi: 10.1016/j.jtos.2021.03.010 (PMC8343164; doi:10.1016/j.jtos.2021.03.010)
Supplement: Multimedia component 14 [file mmc14.docx]

| **ANTIDOBY** | **SPECIES** | **dilution factor** | **Company** | **CAT. Number** |
| --- | --- | --- | --- | --- |
| Anti-periostin (POSTN) | rb | 1:100 | ABCAM | AB14041 |
| Anti-LYPD2 | rb | 1:200 | biorbyt | orb33258 |
| Anti-OGN | rb | 1:50 | Sigma | HPA013132 |
| Anti-KERATIN 3 | ms | 1:50 | Millipore | CBL218 |
| Anti-KERATIN 4 | rb | 1:100 | ABCAM | AB51599 |
| Anti-KERATIN 6A | rb | 1:500 | Biolegend | 905702 |
| Anti-KERATIN 12 | rb | 1:100 | ABCAM | AB185627 |
| Anti-KERATIN 13 | rb | 1:500 | ABCAM | AB92551 |
| Anti-KERATIN 15 | rb | 1:50-500 | ABCAM | AB52816 |
| Anti-S100A8/A9 COMPLEX | rb | 1:100 | ABCAM | AB22506 |
| Anti-GPA2(G-3) | ms | 1:100 | Santa Cruz | SC-390194 |
| Anti-GPA2(G-3) conjugated Alexa 488 | ms | 1:50 | Santa Cruz | SC-390194-AF488 |
| Anti-Amphiregulin (AREG) | rb | 1:100 | Invitrogen | PA5-27298 |
| Anti-Fibulin (FBLN) | ms | 1:200 | Invitrogen | MA5-24598 |
| Anti-Claudin4 | ms | 1:200 | Invitrogen | 32-9400 |
| Anti-FOXC2 | gt | 1:100 | ABCAM | AB-5060 |
| Anti-MelanA | rb | 1:100 | ABCAM | AB51061 |
| Anti-MUC4 | ms | 1:200 | ABCAM | AB60720 |
| Anti-MMP3 | rb | 1:50 | ABCAM | AB52915 |
| Anti-MT2A | ms | 1:100 | Sigma-Aldrich | SAB1402848 |
| Anti-CX30 (GJB6) | rb | 1:200 | ABCAM | AB59927 |
| Anti-CDH19 | rb | 1:200 | ABCAM | AB185573 |
| Anti-CCL21 | ms | 1:200 | ABCAM | AB89396 |
| Anti-α smooth muscle Actin (ACTA2) | rb | 1:200 | ABCAM | AB5694 |
| Anti-TAGLN | gt | 1:100 | ABCAM | AB10135 |
| Anti-CPVL | rb | 1:200 | ABCAM | AB204553 |
| Anti-TFPI2 | rb | 1:50 | Origene | TA324268 |
| Anti-CD105 | ms | 1:200 | ABCAM | AB11414 |
| Anti-Hemoglobin subunit α (HBA1) | rb | 1:100 | ABCAM | AB215919 |
| Anti-CD11c | rb | 1:100 | ABCAM | AB52632 |
| Anti-Ki67 | rb | 1:200 | ABCAM | AB15580 |
| Anti-Ki67 | ms | 1:100 | Millipore | MAB4190 |
| Anti-Ki67 | ms | 1:50 | B&D | 550609 |
| Anti-MITF | rb | 1:200 | ABCAM | AB20663 |
| Anti-CD34 | rb | 1:200 | ABCAM | AB198395 |
| Anti-MMP1 conjugated Alexa 546 | ms | 1:50 | SANTA CRUZ | SC-21731 |
| Anti-MMP1 conjugated Alexa 647 | rb | 1:50 | Abcam | AB196905 |
| Anti-MMP10 | rb | 1:500 | ABCAM | AB59437 |
| Anti-CPVL (H-7) | ms | 1:50 | Santa Cruz | SC-376658 |
| Anti-CD8 | rat | 1:50 | ABCAM | AB22378 |
| Anti-CCL21 | gt | 1:200 | R&D | AF366 |
| Anti-P63 | ms | 1:40 | ABCAM | AB735 |
| Anti-P63 | rb | 1:50-100 | Cell Signaling Technology | 4892 |
| Anti-P63 (P40) | rb | 1:200 | Novus Biologicals | NBP2-29467 |
| Anti-p27 | ms | 1:50 | SANTA CRUZ | SC-1641 |
| C/EBP δ | ms | 1:50 | SANTA CRUZ | SC-365546 |
| Anti-Lumican | ms | 1:50 | SANTA CRUZ | SC-166871 |
| Anti-Lumican | rb | 1:250 | ABCAM | AB168348 |
| Anti-LYVE1 | rb | 1:100 | ABCAM | AB14917 |
| Anti-CD200 (Otx2) | gt | 1:200 | R&D | AF1979 |

**Table S14:** **List of antibodies used for the IHC.**
