# Supplementary material for: A single cell atlas of human cornea that defines its development, limbal progenitor cells and their interactions with the immune cells
Source: Ocul Surf. 2021 Jul;21:279–98. doi: 10.1016/j.jtos.2021.03.010 (PMC8343164; doi:10.1016/j.jtos.2021.03.010)
Supplement: Multimedia component 15 [file mmc15.docx]

**Table S15:** **List of primers used for the qRT-PCR ANALYSIS.**

| Gene | Forward Primer  Sequence (5'->3') | | Reverse Primer  Sequence (5'->3') |
| --- | --- | --- | --- |
| *KRT3* | CGTACAGCTGCTGAGAATGA | CTGAGCGATATCCTCATACT | |
| *KRT12* | GAAGAAGAACCACGAGGATG | TCTGCTCAGGGATGGTTTCA | |
| *KRT14* | TTCTGAACGAGATGCGTGAC | GCAGCTCAATCTCCAGGTTC | |
| *KRT15* | CATGCGCTCTATTCCCCCTC | AGATCAGAGGCCAACCAGGA | |
| TP63 | CTGGAAAACAATGCCCAGAC | GGGTGATGGAGAGAGAGCAT | |
| *GAPDH* | TGCACCACCAACTGCTTAGC | GGCATGGACTGTGGTCATGAG | |
| *GPHA2* | AAACTGCAAGCCGCTCTGTTC | GCAGATAGAGGACCAGGGTTTG | |
| *MUC1* | TCTCACCTCCTCCAATCAC | GAAATGGCACATCACTCAC | |
| *MUC4* | CTTACTCTGGCCAACTCTGTAGTG | GAGAAGTTGGGCTTGACTGTC | |
